# Nucleobase pairing and photodimerization in a biologically derived metal-organic framework nanoreactor

**DOI:** 10.1038/s41467-019-09486-2

**Published:** 2019-04-08

**Authors:** Samantha L. Anderson, Peter G. Boyd, Andrzej Gładysiak, Tu N. Nguyen, Robert G. Palgrave, Dominik Kubicki, Lyndon Emsley, Darren Bradshaw, Matthew J. Rosseinsky, Berend Smit, Kyriakos C. Stylianou

**Affiliations:** 1 Laboratory of Molecular Simulation (LSMO), Institut des Sciences et Ingénierie Chimiques (ISIC), École Polytechnique Fédérale de Lausanne (EPFL Valais Wallis), Rue de l’Industrie 17, CH-1951 Sion, Switzerland; 2 Department of Chemistry, University College London, 20 Gordon St, London, WC1H 0AJ UK; 3 Laboratory of Magnetic Resonance (LRM), Institut des Sciences et Ingénierie Chimiques (ISIC), École Polytechnique Fédérale de Lausanne (EPFL), CH-1015 Lausanne, Switzerland; 4 School of Chemistry, University of Southampton, Highfield Campus, Southampton, SO17 1BJ UK; 5 Department of Chemistry, University of Liverpool, Crown Street, Liverpool, L69 7ZD UK

## Abstract

Biologically derived metal-organic frameworks (bio-MOFs) are of great importance as they can be used as models for bio-mimicking and in catalysis, allowing us to gain insights into how large biological molecules function. Through rational design, here we report the synthesis of a novel bio-MOF featuring unobstructed Watson-Crick faces of adenine (Ade) pointing towards the MOF cavities. We show, through a combined experimental and computational approach, that thymine (Thy) molecules diffuse through the pores of the MOF and become base-paired with Ade. The Ade-Thy pair binding at 40–45% loading reveals that Thy molecules are packed within the channels in a way that fulfill both the Woodward-Hoffmann and Schmidt rules, and upon UV irradiation, Thy molecules dimerize into Thy<>Thy. This study highlights the utility of accessible functional groups within the pores of MOFs, and their ability to ‘lock’ molecules in specific positions that can be subsequently dimerized upon light irradiation, extending the use of MOFs as nanoreactors for the synthesis of molecules that are otherwise challenging to isolate.

## Abstract (web summary)

Metal-organic frameworks have shown promise as nanoreactors, facilitating the synthesis of molecules that are otherwise difficult to isolate. Here, the authors design a framework featuring unobstructed adenine linkers to which thymine molecules can base-pair, allowing for thymine dimerization in the pores upon UV irradiation.

## Introduction

The structural versatility, permanent porosity, tunable pore surfaces, and periodic nature of metal–organic frameworks (MOFs) provide a unique opportunity to study their potential in applications ranging from gas storage and separation, to UV-induced coupling, catalysis, and sensing^1–7^. Biologically-derived MOFs (bio-MOFs) based on ligands such as amino acids, nucleobases, and oligosaccharides have shown a great potential in many research areas as they can be used as models for bio-mimicking and catalysis^8,9^. There is a significant interest in observing biological phenomena in porous bio-MOFs, as we can gain insights into their response upon the inclusion of guest molecules and how guest molecules behave within these confined spaces^8–15^. These insights can ultimately lead into the generation of novel bio-MOFs with tunable pore functionalization, allowing one to target specific chemical reactions and isolate desired products^16–19^. This has the potential to open up a new field of research related to drug design, discovery, delivery, and catalysis^20,21^.

Exploiting functional pore surface in MOFs has recently been utilized in carbohydrate separation and labeling and photochemical transformations^22–24^. Intrigued by the pore surface interactions with guest molecules, and the use of functional groups as structure-directing agents, herein we investigated the Watson–Crick (W–C) face of adenine (Ade) within a new MOF (hereafter called as SION-19) for nucleobase pairing with thymine (Thy), and solid-state photodimerization of Thy to Thy<>Thy, a molecule that is related to skin cancers such as melanoma^25^. For this concept, two prerequisites govern both the ability of the MOF to act as a nanoreactor, and the ability of Thy to undergo dimerization. First, the strength of Ade–Thy nucleobase pairing within the pores of the MOF is determined by its pore shape and size. Thy not only needs to be able to fit within the pore, but an optimal distance of <3 Å between Ade–Thy is required to allow for the formation of H-bonds via the W–C face of Ade^26^. Here, the structure directing ability of Ade within SION-19 can ‘lock’ Thy into a position close enough to another Thy for photodimerization to occur. Second, for successful dimerization, both the Woodward–Hoffmann and Schmidt rules would have to be fulfilled^27–29^. This implies that the C5–C6 and C5′–C6′ double bonds of Thy (according to its conventional atom numbering scheme) have to be <4.2 Å apart (center-to-center)^29^ and the molecular orbital symmetry has to be conserved^28^. Ultimately, an overall balance between these requirements is needed, as pores too large or of improper shape can disfavor Ade–Thy base-pair interaction, while incorrect packing of Thy within the pores can affect their ability to dimerize due to poor orientation of the C5–C6 and C5′–C6′ double bonds.

MOFs with unobstructed W–C faces of Ade reported in the literature are scarce and the generation of materials with a precise pore aperture for Thy uptake has proven to be challenging^17,30,31^. In addition, the dimerization of Thy and formation of Thy<>Thy has only been achieved either in liquid systems such as freezing water, in the presence of sensitizers such as acetone or when Thy is ordered in DNA struts^32–34^. Inspired by this challenge, and in order to highlight the structural tunability of MOFs, we demonstrate for the first time, that Ade–Thy binding can be achieved within the pores of SION-19, and Thy can be dimerized to Thy<>Thy in solid state at 40–45% Thy-loadings.

## Results

### Crystal structure determination of SION-19

**Fig. 1:**
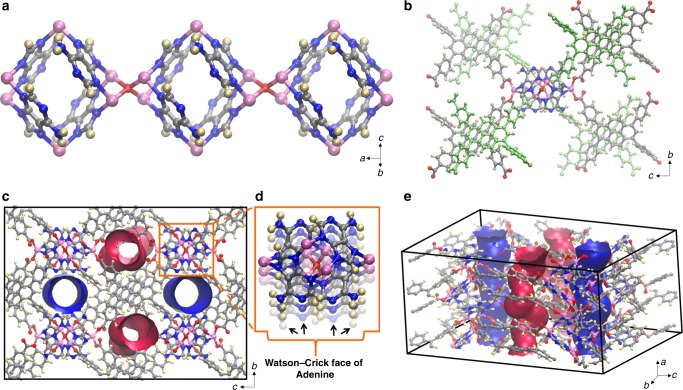
Structural representation of SION-19. **a** A Zn^II^(adeninate) octahedron cage is coordinated to six Zn^II^ ions, four at the corners of the equatorial plane (Zn^II^_eq_) and two at the apical positions (Zn^II^_ap_). The Zn^II^(adeninate) cages are linked to each other via the Zn^II^_eq_ to form 1-dimensional columnar building blocks. **b** The Zn^II^(adeninate) columnar building blocks are linked to each other via fully deprotonated TBAPy ligands—gray TBAPy is bound to Zn^II^_ap_ whereas the lime green is bound to Zn^II^_eq_. **c**, **d** Connolly surface representation of SION-19 viewed along the *a*-axis, revealing that the coordination of TBAPy ligands to the Zn^II^(adeninate) columns leads in the formation a 3-dimensional structure possessing two distinct types of 1-dimensional pores: the base-pore (blue) and the acid-pore (red). Highlighted in (**d**) are the Watson–Crick faces of Ade which point into the base pore and **e** Connolly surface representation of SION-19 in a 3-dimensional view. Atom color code: pink, Zn; red, O; blue, N; gray, C; light yellow, H

The reaction of Zn(NO_3_)_2_·6(H_2_O), 1,3,6,8-tetrakis(*p*-benzoic acid) pyrene (H_4_TBAPy)^35,36^ and Ade in a solvent mixture of DMF/H_2_O/HNO_3_ at 120 °C for 72 h leads to the generation of yellow truncated rhombic bipyramid crystals. Single-crystal X-ray diffraction (SCXRD) reveals that anionic SION-19, with formula of [Zn_1.5_O_0.25_(Ade)(TBAPy)_0.5_](NH_2_Me_2_)_0.5_·(DMF)_0.6_·(H_2_O)_4.0_, crystallizes in an orthorhombic unit cell with the *Ccce* space-group symmetry and cell parameters of *a* = 10.7402(3) Å, *b* = 30.6236(7) Å, *c* = 42.6282(11) Å, *α* = *β* = *γ* = 90° with *V* = 14020.6(6) Å^3^ (Supplementary Discussion 2-3). Within SION-19, octahedral cages are constructed by four Ade ligands and six Zn^II^ tetrahedral ions, four of which are in the equatorial plane (Zn^II^_eq_) and two at the apical positions (Zn^II^_ap_) (Fig. 1a). The Zn^II^_eq_ link the cages by Zn_4_O clusters forming the adeninate columnar building units running along the *a*-axis. The tetrahedral geometry of Zn^II^_eq_ is provided by the N3 and N9 atoms from Ade, one μ_4_-O bridge and one carboxylate O from a TBAPy ligand. For Zn^II^_ap_, the tetrahedral coordination environment is owed to two N7 atoms from two adjacent Ade ligands, as well as two monodentate carboxylate O atoms from two consecutive TBAPy ligands (Fig. 1b). Within SION-19, Ade acts as a deprotonated and bridging tridentate ligand, while the fully deprotonated and disordered TBAPy ligand coordinates in a monodentate fashion from each carboxylate (Supplementary Figure 2). The TBAPy ligands, inclined with respect to the *a*-axis, link the Zn^II^-based columnar units to yield a structure expanding in three dimensions. However, due to the geometrical features of these building units and TBAPy, infinite channels endowed with two markedly different chemical character are formed along the *a*-axis. These pores are classified as the acid-pore and the base-pore (Fig. 1c, d). The acid-pore in which the free carboxylate O atoms from the TBAPy ligand are pointing in, has a pore dimension of 7.9 Å × 4.9 Å (including van der Waals radii), while the base-pore contains the unobstructed W–C faces of Ade and its dimensions are 5.4Å × 6.9 Å (Fig. 1e). Four Ade ligands attached to the same Zn_4_O cluster point to four different channels, while two Ade linked to the same Zn^II^_ap_ ion are arranged in the antiparallel fashion; therefore, even though all Ade are grouped relatively close to each other within the crystal structure, their spatial orientation allows for all of their W–C faces to be exposed to the channels. These pores, in turn, are occupied by disordered guest solvent molecules and cations that show no indication of preferred location. The accessible volume of SION-19 is 31.3%, and combining the accessible volume with the density of the static structure results in a pore volume of 0.287 cm^3^/g. Topological analysis of SION-19 using the TOPOS4.0 software package reveals a new 4,4,8-c net with the point symbol {4^2^·6^4^}4{4^4^·6^14^·8^10^}^37^. This net has been registered in the Topos Topological Database (TTD) collection as *kcs1* (Supplementary Figure 3).

### Bulk characterization

**Fig. 2:**
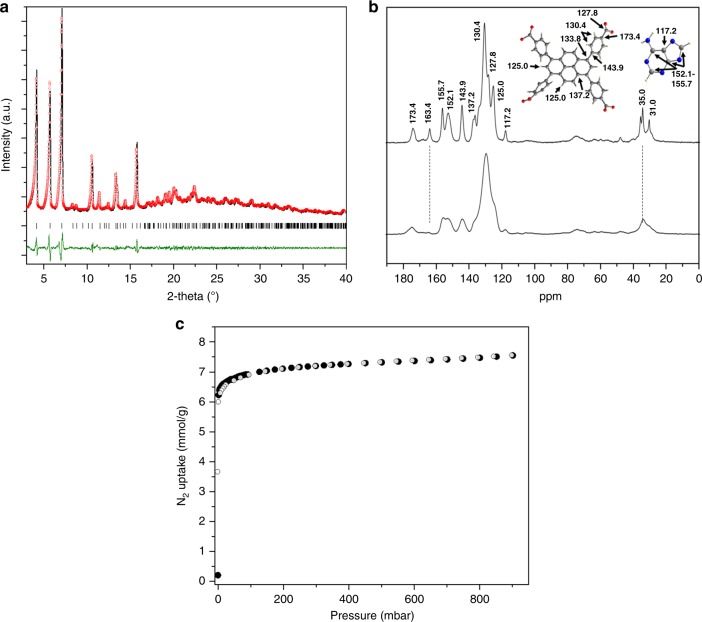
Solid-state characterization of SION-19 and SION-19′. **a** Final fit (Rietveld refinement) for a rigid-body structural refinement of the PXRD pattern of SION-19 (final observed: red circles, calculated: solid black lines, and difference: solid green line). **b** Solid-state ^13^C CP MAS NMR spectra of (top) SION-19 and (bottom) SION-19′ collected at 298 K. The assignment of the resonances in SION-19 is consistent with the expected resonances corresponding to the TBAPy and Ade ligands as well as to the DMF and [NH_2_Me_2_]^+^ molecules residing within the channels. **c** Type I N_2_ isotherm measured on SION-19′ at 77 K and 1 bar (filled symbols: adsorption, empty: desorption)

Powder X-ray diffraction (PXRD), elemental analysis (EA), thermogravimetric analysis (TGA) (Supplementary Discussion 4.1), and solid state ^13^C CP/MAS NMR confirmed the purity and the structural stability of SION-19. Le Bail fit of the PXRD pattern of SION-19 collected at 298 K gives refined cell parameters of *a* = 10.965(7) Å, *b* = 30.811(2) Å, *c* *=* 42.261(6) Å, *α* = *β* = *γ* = 90°, *V* = 14277.5 Å^3^ (*Ccce* space-group symmetry, and fit indicators: *R*_wp_ = 4.7% and *χ*^2^ = 1.9) which are in good agreement with the cell parameters obtained by SCXRD (Fig. 2a). The EA revealed that the guest solvent molecules comprise 26.9% of the mass of SION-19, which is consistent with the TGA mass loss of 27.9%. SION-19 is stable up to 450 °C. Full activation of SION-19 can be achieved upon heating at 110 °C under dynamic vacuum (10^−6^ mbar) for 12 h, giving rise to SION-19′ (Supplementary Discussion 4.2). SION-19′ is stable in DMF, EtOH, and CH_3_CN solutions, and both as made and desolvated materials are bench top stable for extended periods of time (Supplementary Figure 10). As can be seen in Supplementary Figure 11, SION-19′ loses its crystallinity to a significant degree but its PXRD pattern can still be indexed with cell parameters *a* = 11.048(9) Å, *b* = 31.268(9) Å, *c* = 43.140(1) Å, *α* = *β* = *γ* = 90°, *V* = 14902.6 Å^3^, *Ccce* space-group symmetry, and fit indicators of *R*_wp_ = 4.8% and *χ*^2^ = 2.3. Both EA and X-ray photoelectron spectra (XPS) collected on H9Ade, [(Me)_2_NH_2_]Cl and SION-19′ confirm the presence of [NH_2_Me_2_]^+^ in the pores (Supplementary Discussion 5) which were unable to be directly observed through SCXRD or TGA analyses^38^. To further resolve the location of cations within the pores of SION-19, we performed classical molecular dynamics (MD) simulations which show a clear preference for the [NH_2_Me_2_]^+^ to localize near the carboxylate groups in the acid-pores of SION-19. This is further supported by the time-averaged energy difference of 14 kcal/mol/cation in favor of the acid-pore over the base-pore (Supplementary Figure 14).

In order to probe any structural changes upon full activation of SION-19, ^13^C CP/MAS NMR was utilized. The complete removal of the guest molecules from SION-19 is confirmed by (i) the broadening of the C-signals corresponding to the TBAPy and Ade ligands and (ii) the absence of the carbonyl carbon of DMF at 163.4 ppm (Fig. 2b). The NMR spectrum of SION-19′ shows that aliphatic carbons of [NH_2_Me_2_]^+^ are still present at 31.0 and 35.0 ppm as broad peaks. Combining our findings, it is apparent that the structural backbone of SION-19 is maintained when is activated to SION-19′.

### Sorption properties of SION-19′

The permanent microporosity of SION-19′ was demonstrated by the type I N_2_ adsorption isotherm collected at 77 K and 1 bar (Fig. 2c), and it was also found that SION-19′ is porous to CO_2_ and CH_4_ at 273 or 298 K and 1 bar (Supplementary Figure 13). The BET surface area and Dubinin–Radushkevich pore volume of SION-19′ were calculated to be 562(8) m^2^/g and 0.246 cm^3^/g, respectively. These values are in agreement with the molecular simulations, when [NH_2_Me_2_]^+^ species are placed in the acid pores of SION-19′ (Supplementary Discussion 7.1). Dynamic N_2_-probed properties of this simulation report an average void volume of 0.213 cm^3^/g (Supplementary Figure 15) and a surface area of 672 m^2^/g (Supplementary Figure 16). This also confirms the presence of [NH_2_Me_2_]^+^ in the acid-pore and thus they will not compete with other guest molecules for Ade binding in the base pore.

### Thymine loading and dimerization

**Fig. 3:**
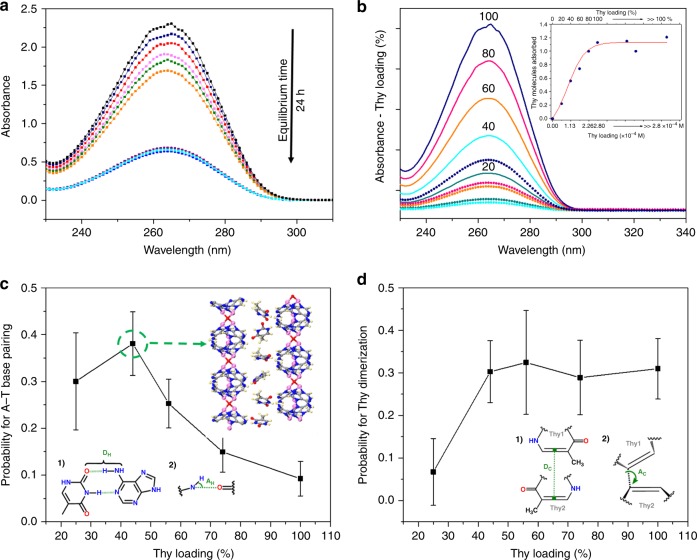
Solution thymine isotherms in SION-19′ and probability of adenine–thymine pairing and thymine-dimerization. **a** Time dependency for Thy to reach equilibrium when loaded into SION-19′. **b** Solution isotherms of 20–100% (0.56–2.80 × 10^−4^ M) Thy loaded into the pores of SION-19′. Inset: maximum uptake of Thy (~1.1 molecules). Here, multiple Thy concentrations (blue circles—above 100% loading) were used to determine the saturation limit of Thy into the pores of SION-19′. **c** Frequency of observed Ade–Thy base pair formation from MD simulations—Insets (1) and (2) show the metrics used for H-bonding formation between Ade–Thy from the MD simulations. **d** The frequency in which Woodward–Hoffmann and Schmidt rules are satisfied for Thy dimerization in the MD simulations at different loadings. Insets (1) and (2) show the metrics used for satisfying the Woodward–Hoffmann and Schmidt rules

Based on the structural uniqueness of SION-19 and accessibility of the W–C face of Ade pointing towards the base-pore, room temperature Thy solution isotherms were performed in a mixture of EtOH:MeCN solution (20:80) with loadings ranging from 20 to 100%. These loadings were calculated based on the accessible volume of each formula unit in SION-19 (242.0 Å^3^) and the molecular volume of Thy (142.2 Å^3^). Following a loading of 100%, the equilibrium was established via UV/vis spectroscopy to be for 24 h (Fig. 3a, b)^39^. SION-19′ can uptake ~1.1 molecules of Thy at 100% loading (Fig. 3b, inset), and this is in agreement with the formula derived from EA:[Zn_1.5_O_0.25_(Ade)(TBAPy)_0.5_](NH_2_Me_2_)_0.5_·(Thy)_0.95_·1.1EtOH·1.5H_2_O. Although the crystals of SION-19′ lose their singularity which precluded the use of SCXRD for the purpose of SION-19@Thy structure determination, the reduced BET surface area: 79(4) m^2^/g (Supplementary Figure 22), FT-IR (free Thy carbonyl at 1702 cm^−1^, and at 1699 cm^−1^ when present in SION-19), and solid state ^13^C MAS NMR and ^15^N CP NMR, which showed Thy shifts in SION-19@Thy of δ 156.1701 ppm (N3), and δ 126.5180–130.8505 ppm (N1) (Supplementary Discussion 7.2-7.3) confirmed the diffusion of Thy within the pores of SION-19′. Using DFT calculations, EtOH molecules were simulated in the pores of SION-19′ and shown to preferentially reside in the acid pores (where no Ade–Thy H-bonding can occur) by 3.3 kcal/mol. This is due to favorable interactions with the charge-balancing cations [NH_2_Me_2_]^+^ present in the acid pores.

The nature of the Ade–Thy base pair formation in SION-19′ was examined through DFT optimization of one Thy near Ade^17^. The optimized distances between H-bonding donors and acceptors are 2.72 Å (N_Ade_–N_Thy_) and 3.00 Å (N_Ade_–O_Thy_), and are comparable with the experimentally measured distances of 2.82 and 2.95 Å, respectively^40^. In addition, Bader population analysis shows a slight charge polarization of 0.06 e^−^ on the heavy atoms^41^, indicative of H-bonding formation between Thy and Ade in SION-19′. The DFT binding energy for a single Thy in SION-19′ is calculated to be −18.2 kcal/mol, which has higher absolute value compared to the ~−12 kcal/mol binding energy reported for pure Ade–Thy interactions in both experimental and theoretical calculations^42,43^. Dynamic, finite temperature behavior of Thy within the pores of SION-19′ was investigated with classical MD simulations at room temperature (298 K). In these simulations, base-pair H-bonding was modeled using three-bodied potentials using parameters designed for accurate H-bonding in bio-molecules^44^. With this potential, we observe a good energetic agreement with the DFT binding energy computed for Ade–Thy in SION-19′, being 1.4 kcal/mol higher in energy (−19.6 vs −18.2 kcal/mol).

**Fig. 4:**
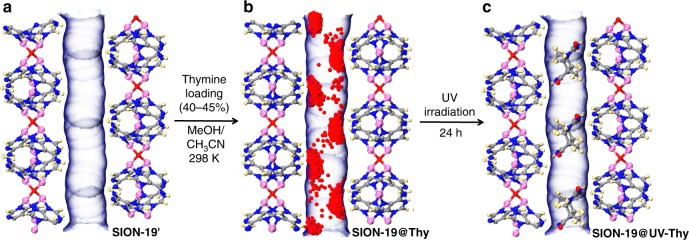
Schematic representation of thymine loading, location, and dimerization within SION-19′. **a** 1-dimensional base-pore surface for SION-19′ prepared using HOLE2. **b** Thy snapshots (red dots) from an MD trajectory of 44% thymine loading in the base pore of SION-19. Here, Ade acts as a structure directing agent that ‘lock’ Thy molecules into positions close enough to another Thy molecule for photodimerization to occur. **c** DFT optimized positions of Thy<>Thy dimers arising from a 44% loading of Thy. Atom color code: pink, Zn; red, O; blue, N; gray, C; light yellow, H

Exposure of pyrimidine nucleobases like Thy to UV light can induce a [2 + 2] cycloaddition between the C5–C6 and C5′–C6′ double bonds of two pyrimidine rings (Supplementary Scheme 1)^33^. While dimerization of Thy in a liquid medium is a viable method to afford Thy<>Thy, its solid-state dimerization has not been fully studied. To confirm this, Thy crystals were exposed to UV light (254 nm) for 24 h and ^1^H NMR revealed that no Thy<>Thy was obtained (Supplementary Figure 27). To investigate the atomistic features of the Thy loading experiments in SION-19′, room temperature MD simulations were performed at loadings of 25, 44, 56, 74, and 100%. Due to the relatively tight pores, and the strong H-bonding potentials used in the simulation, 5 cycles of 2 ns annealing were performed at each loading. Statistics on the orientations of Thy with respect to themselves, and Ade in SION-19′ were collected over the span of 10 ns at intervals of 2 ps. It was hypothesized that in order to enable the formation of Thy<>Thy, the Thy molecules must resemble the transition state of a [2 + 2] cycloaddition, and follow the Schmidt rules. That is, the C5–C6 and C5′–C6′ must be nearly parallel, and separated by <4.2 Å^29^. Thus, the trajectories for the potential formation of a four-membered ring were analyzed, specifically when the C5–C6 double bond of adjacent Thy molecules were (i) within 4.2 Å of each other, and (ii) nearly aligned, by determining the dihedral angle formed between C5–C6 and C5′–C6′ is below 30°^29^. Figures 3c, d and 4 demonstrate that Thy molecules appear to be more favorably oriented for dimerization at 44% loading and above. This is compared to the peak in base-pair formation, which occurs at a 44% loading and then subsequently deteriorates. At higher Thy loadings, Thy packs in a dense sterically unfavorable manner within the base pore disfavoring base-pair formation (Fig. 3c). This correlation suggests that the Ade ligands within SION-19′ are structure-directing agents, which fix the position of Thy within the pores, and can allow for Thy<>Thy formation from two nearby ‘locked’ base-paired Thy molecules. A plot of the trajectories at a 44% loading show that Thy molecules form base pairs with Ade that are two Ade units apart along the *a*-axis, and on opposite sides of the pore (Fig. 3d). To validate this experimentally, samples of SION-19@Thy(20–80%) were continuously exposed to UV for 24 h^45^. The formation of Thy<>Thy was determined via ultra-high performance liquid chromatography coupled with electrospray ionization mass spectrometry (UHPLC-ESI/MS) (Supplementary Discussion 8.4-8.4.1). Samples of SION-19@UV-Thy(20–30, 50–80%) (Supplementary Figures 35) showed no presence of product, while SION-19@UV-Thy(40–45%) (Supplementary Figure 36-41) afforded Thy<>Thy in a non-quantitative yield (58.5%). The non-quantitative nature of this dimerization is thought to be due (i) to the low population of an excited state (singly excited, ^1^SE), which proceeds through an internal conversion, and requires overcoming an energy barrier^27^, and (ii) to the poor penetration of light throughout the solid material, and thus some Thy molecules do not have enough energy to overcome this barrier and dimerize. Here, this mechanism is governed by both conformational control, and electronic effects^27–29^. The inability of lower Thy loadings (20–30%) to dimerize might be due to the increased spatial distance between Thy molecules as they slowly diffuse through the channels, while at higher loadings (50–80%), there is a decrease of H-bonding stabilization between the framework Ade and Thy, leading to an unfavorable packing within the base-pore.

## Discussion

To further confirm the structural uniqueness of SION-19′ to ‘lock’ Thy into a specific orientation and transfer energy for dimerization, Thy loading and dimerization in the pores of adenine-containing bio-MOF-1 as well as HKUST-1 and Zn-MOF-74, which are not adenine-based, were attempted, and in all cases no traceable amounts of Thy<>Thy were observed (Supplementary Discussion 9). These results further confirm that not only the presence of the W–C faces of Ade, but also the optimal Ade–Thy binding ratio and the subsequent electronic effects/conformation control, play an important role in the dimerization process. That is, the stabilization of Thy via W–C H-bonding with Ade in SION-19 allows it to conform to both the Schmidt and Woodward–Hoffmann rules.

In conclusion, we synthesized a novel biologically derived MOF, SION-19, possessing a new *kcs1* topology and Ade-functionalized pore surface, that acts as a nanoreactor for the dimerization of Thy via site-specific binding to Ade. We successfully showed that the orientation of Ade within SION-19 permits Thy binding (40–45% loading) that resembles the transition state of [2 + 2] cycloaddition required for Thy dimerization. Thy loading and dimerization in the pores of SION-19, and the specific relationship between Ade–Thy binding demonstrate that taking advantage of functional groups that decorate the pore surface of MOFs can be used as an effective strategy for their utilization as nanoreactors for photo-induced organic reactions.

The ability to photo-induce the dimerization of solid state Thy within the constrained pores of SION-19 can ultimately provide new fundamental insights on the governing mechanism of this process. Our approach can be applied to a variety of MOFs or other porous materials with comparable structural features to ‘lock’ molecules in specific positions, and can open up new avenues for the synthesis of organic molecules that may otherwise be difficult to obtain through traditional routes. Furthermore, using bio-MOFs as nanoreactors to form biologically relevant molecules can bridge multiple research disciplines, which can aid in the development of new methods for drug discovery and delivery. These strategies can offer an opportunity for interdisciplinary work, that is, to develop new synthetic methodologies and study the activity of new bio-related products.

## Methods

### Synthesis of SION-19, thymine loading, and photodimerization

Reagents and solvents were purchased from Sigma-Aldrich (Zn(NO_3_)_2_·6(H_2_O)), TCI (Ade), and Carl Roth (DMF) and used without further purification.

SION-19 was synthesized through the combination of Zn(NO_3_)_2_·6(H_2_O) (17 mg, 0.057 mmol), TBAPy^35^ (10 mg, 0.0146 mmol) and Ade (8 mg, 0.059 mmol), in an acidic DMF:H_2_O:HNO_3_ (5.5 mL:0.5 mL:4 drops). The vial is then capped and placed in the oven for 72 h at 120 °C with a temperature heating ramp of 2.0 °C, and cooling ramp of 0.2 °C, affording yellow colored truncated rhombic bipyramid like crystals in a 28% yield (based off of Zn). The formula of the 3D framework of SION-19 was determined to be [Zn_1.5_O_0.25_(Ade)(TBAPy)_0.5_](NH_2_Me_2_)_0.5_·(DMF)_0.6_·(H_2_O)_4.0_ from SCXRD analysis, while TGA and EA allowed for the determination of the pore content. Anal. Calcd for bulk SION-19 sample [Zn_1.5_O_0.25_(Ade)(TBAPy)_0.5_](NH_2_Me_2_)_0.5_·(DMF)_1.7_·(H_2_O)_4.0_: C 49.95, H 5.05, N 12.67. Experimental: C 50.03, H 4.57, N 12.84. Note: the discrepancies between the chemical composition derived from single crystal and bulk analysis might be due to the bulk sample reflecting the average composition of all crystals.

Desolvation of SION-19 was carried out at 110 °C under high vacuum (10^−6^ mbar). The formula of SION-19′ was determined to be [Zn_1.5_O_0.25_(Ade)(TBAPy)_0.5_](NH_2_Me_2_)_0.5_·(H_2_O)_0.2_. EA of SION-19′: Anal. Calcd for [Zn_1.5_O_0.25_(Ade)(TBAPy)_0.5_](NH_2_Me_2_)_0.5_·(H_2_O)_0.2_: C 55.99, H 3.22, N 12.83. Experimental: C 55.78, H 3.13, N 13.19.

Desolvated ground host material (100 mg), SION-19′, was immersed in an EtOH:MeCN (20:80) solution in a Schlenk tube under a N_2_ atmosphere, which contained a known concentration of Thy. The accessible volume per unit cell for SION-19′ is 31.3% as calculated by MERCURY using the probe radius of 1.2 Å and approx. grid spacing of 0.7 Å, therefore the free volume per unit cell is 3872.11 Å^3^. In one unit cell, there are 16 formula units (FU: [Zn_1.5_O_0.25_(Ade)(TBAPy)_0.5_](NH_2_Me_2_)_0.5_), resulting in an accessible volume of 242.0 Å^3^ per FU. The volume of a single Thy molecule, calculated from its crystal structure, was found to be 142.219 Å^[3 46^, and therefore, theoretically 1.70 molecules of Thy can fit per FU of SION-19′. Based on these calculations, the concentration of Thy was varied in order to keep the proportion of filling between 20 and 100% (0.56–2.80 × 10^−4^ M). SION-19′ was also immersed at higher concentrations (up to 7.11 × 10^−4^ M) to check the saturation uptake. Through UV–vis, the time dependency for equilibrium of 100% Thy loading was established to be 24 h. Subsequently, once both the equilibrium time and calibration curve of Thy loaded material were established, SION-19′ was loaded with known concentrations of Thy (0.56–2.80 × 10^−4^ M). The absorption of the supernatant was collected and analyzed by UV–vis to quantify the amount of Thy absorbed. Here, the absorption coefficient of thymine in an EtOH:MeCN solution at 263 nm is 8217 cm^−1^/M which is comparable to that of thymine in H_2_O (7900 cm^−1^/M)^47^.

**Fig. 5:**
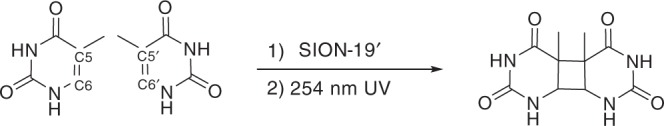
Thymine photodimerization. Synthetic conditions utilized for the photodimerization of Thy molecules using SION-19′

Approximately 50 mg of SION-19@Thy (20–80% loadings of Thy) is exposed to UV continuously for 24 h (Fig. 5). The material was then collapsed in a 6 mL 0.5 M K_2_CO_3_ solution, sonicated for 10 min, diluted with 50 mL of DMSO, and subsequently dried with MgSO_4_, filtered and concentrated using a rotary evaporator^48^. Each sample was dissolved in 2 mL of DMSO and further prepared according to the procedure in Supplementary Discussion 7.4 for UHPLC-ESI/MS.

## Supplementary information

Supplementary Information

## Data Availability

The X-ray crystallographic coordinated for the SION-19 structure reported in this study has been deposited at the Cambridge Crystallographic Data Centre (CCDC), under deposition number 1855564. This data can be obtained free of charge from the Cambridge Crystallographic Data Centre via www.ccdc.ac.uk/data_request/cif.
