# Supplementary material for: Nucleobase pairing and photodimerization in a biologically derived metal-organic framework nanoreactor
Source: Nat Commun. 2019 Apr 8;10:1612. doi: 10.1038/s41467-019-09486-2 (PMC6453978; doi:10.1038/s41467-019-09486-2)
Supplement: Supplementary file 1 — Supplementary Information [file 41467_2019_9486_MOESM1_ESM.pdf]

Nucleobase Pairing and Photodimerization in a Biologically Derived Metal-Organic Framework Nanoreactor

Anderson *et al.*

Supplementary Discussion 1. Experimental: Materials and Characterization Methods

Reagents and solvents were purchased from Sigma-Aldrich, TCI, and Carl Roth and used without further purification. Infrared spectra were collected on a Perkin Elmer FT-IR/FIR Frontier Spectrometer from 400 to 4000 cm^{-1}. Thermogravimetric analysis (TGA) was performed under air atmosphere on a TA instrument SDT Q600. A dried crystalline sample was heated at a rate of 5 ºC/min until 650 ºC and then cooled to room temperature at a rate of 10ºC/min. Powder X-ray diffraction data were collected on a Bruker D8 Advanced using Cu Kα radiation (λ = 1.5418 Å, 50 kW/40mA). Single crystal X-ray diffraction data were collected at the ESRF in Grenoble, France. Simulated powder X-ray diffraction patterns were generated from the single crystal data using Mercury 3.0. MAS ^{1}H-^{13}C NMR spectra were collected on a 400 MHz Bruker NMR, while *insitu* ^{1}H NMR spectra were collected on a 400 MHz Bruker NMR. Elemental analyses (EA) were obtained using a Thermo EA1112 Flash CHNS-O Analyzer. High resolution X-ray photoemission spectra (XPS) on neutral adenine (Ade), commercial available $Me_2NH_2Cl^+$ and **SION-19'** were recorded on a Scienta ESCA 300 spectrometer located at Daresbury Laboratory, UK, which incorporated a rotating anode Al Kalpha (hȞ = 1486.6 eV) X-ray source and has an effective instrument resolution of 400 meV. The spectrometer was calibrated regularly to set the Fermi edge of a silver reference sample at zero binding energy. Samples were mounted on adhesive tape and, to reduce charging, the samples were covered with a stainless steel mask, through which a small area of the sample was exposed. In addition, it was necessary to use an electron flood gun to stabilize the surface charge. Use of a flood gun shifts all spectral features to high kinetic energy, and photoelectron spectra were therefore charge calibrated using a weak C1s contaminant peak (present due to surface hydrocarbon contamination from vacuum pumps and other sources) which was assigned a binding energy of 285.0 eV. The integrity of the samples under the measurement conditions of high vacuum and x-ray irradiation was determined by collecting several sequential N1s region spectra. No changes over time were seen in any sample, suggesting that no significant sample degradation occurred during the measurements. Spectra were fitted using CasaXPS software. All fitted peak shapes were 70:30 Gaussian:Lorentzian convolutions, and linear backgrounds were used in all cases. Gas sorption measurements: Carbon Dioxide, Nitrogen and Methane isotherms were collected on activated **SION-19'** using the Intelligent Gravimetric Analyser Instrument (IGA) from Hiden Isochema. CO_2 isotherms were collected at 195 K, 273 K and 298 K at 1 bar, while N_2 isotherms were collected at 77 K and 1 bar. CH_4 isotherms were measured at 273 K and 298 K at 1 bar. For optical measurements, UV/vis was collected in solid state at room temperature on a Perkin Elmer Lambda 650S UV/vis spectrometer equipped with Labsphere integrating over the spectral range 190–900 nm using $BaSO_4$ reflectance standards. The spectra for **SION-19** and **SION-19'** were collected using the solid-state holder containing with the same amount of solid; the holder for **SION-19'** was filled in the dry box. Fluorescence excitation and emission spectra and lifetimes were collected at room temperature on a Perkin-Elmer LS 55 fluorescence spectrometer. Excitation and emission spectra of **SION-19** and **SION-19'** (sealed in a capillary to prevent any re-absorption of H_2O moisture from the atmosphere from **SION-19'**) were collected in the range between 200-600 nm

and 400-700 nm respectively with scan speed of 10 nm/min. UHPLC was carried out using a Acquity UPLC HSS T3, 1.8 um, 2.1 x 100 mm column from Waters, on a Thermoscientific Orbitrap Q Exactive HF equipped with H-ESI and AP-MADLI source.

Supplementary Discussion 2. Single-Crystal X-ray Diffraction and Topology.

A high-quality single crystal of **SION-19** was isolated from the mother liquor, and mounted onto the PILATUS@SNBL diffractometer at the beamline BM01 of the European Synchrotron Radiation Facility, Grenoble, France. The crystal was probed with X-rays (λ = 0.7519 Å), intensities of Bragg reflections were recorded with the PILATUS2M detector, and the crystal was kept at 100(2) K during the data collection. Raw data were processed with CrysAlisPro (2015) program suite,[1] and the empirical absorption correction was performed using spherical harmonics, implemented in SCALE3 ABSPACK scaling algorithm.

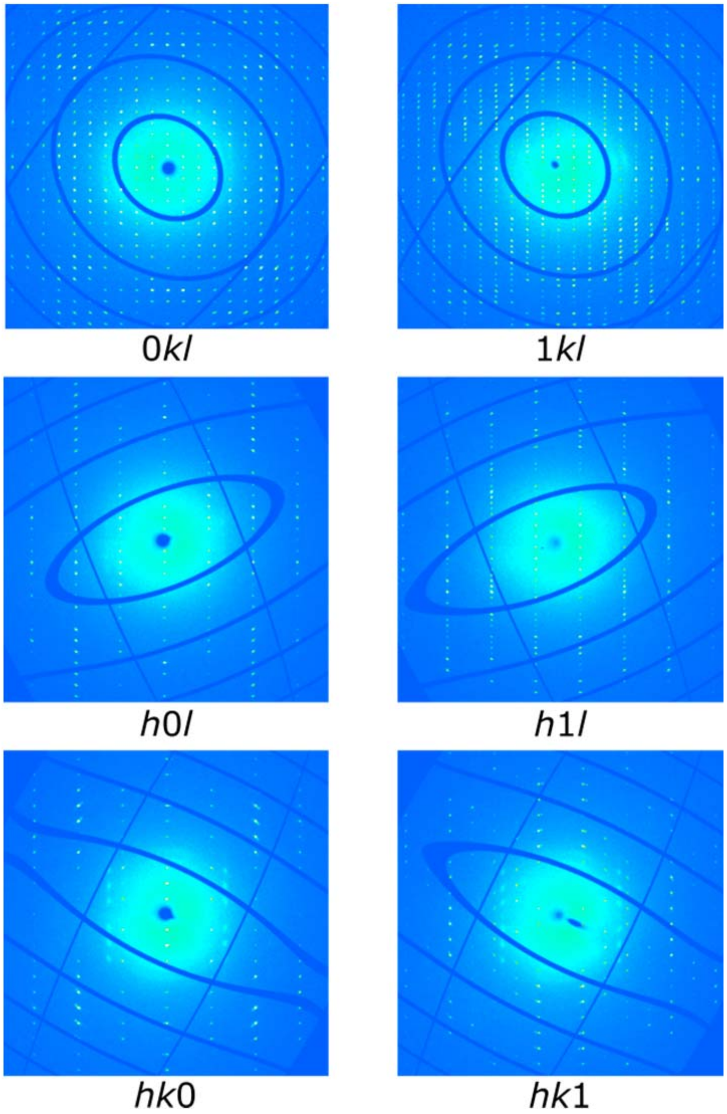

Supplementary Figure 1: Precession photographs reconstructed from the **SION-19** diffraction data. Orthorhombic metric symmetry is confirmed. Systematic absences for integer *n*: 0*kl*: *k* = 2*n*, *l* = 2*n*; *h*0*l*: *h* = 2*n*, *l* = 2*n*; *hk*0: *h* = 2*n*, *k* = 2*n*; *hkl*: *h* + *k* = 2*n*. Extinction symbol *Ccc*(*ab*) corresponds uniquely to the space group *Ccce*.

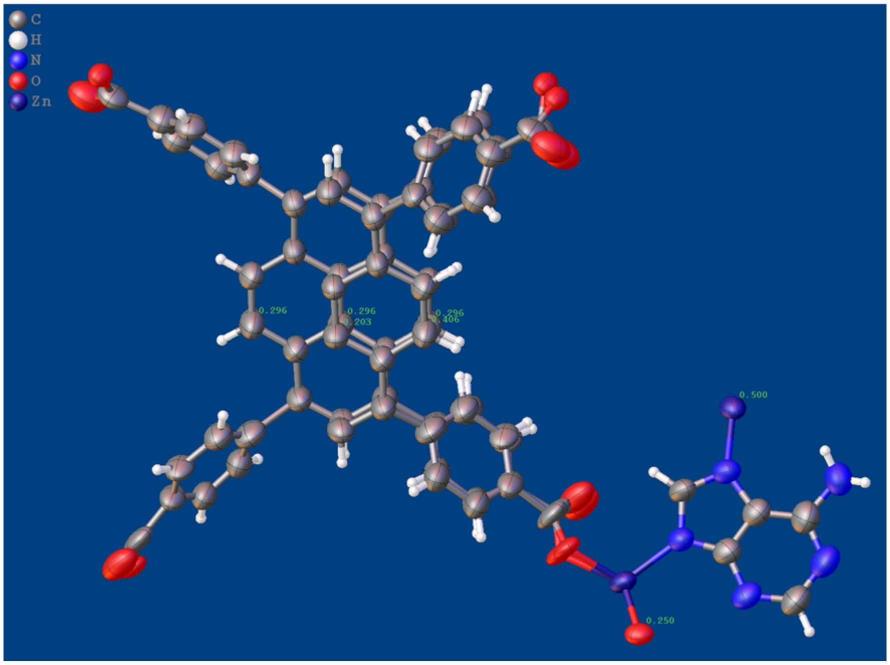

Supplementary Figure 2: Asymmetric unit of **SION-19**. Crystallographic occupancy is shown next to selected atoms. In the TBAPy^{4-} ligand the occupancies are identical within the same parts of disorder. Four C atoms of the half TBAPy^{4-} with 0.406-occupancy that form the long diagonal of the pyrene core lie exactly on the crystallographic 2-fold axis, which reduces their occupancy by a factor of 2.

Crystal structure of **SION-19** was solved with the SUPERFLIP structure solution program using Charge Flipping and refined with the ShelXL refinement package using least-squares minimization,[2-5] implemented in the Olex2 program suite.[6] The solution confirmed the *Ccce* space-group symmetry, observed equally in the reciprocal space reconstruction images (Supplementary Figure 1). Atomic positions were found from the electron density maxima and refined anisotropically for all non-H atoms. In the asymmetric unit (ASU) the following structural units were localized: two Zn(II) atoms, with one on them lying on a special position with 0.5-occupancy, one deprotonated Ade^{-} molecule, one O^{2-} ion lying on a special position with 0.25-occupancy, and half the TBAPy^{4-} ligand. In the crystal structure of **SION-19** the TBAPy^{4-} ligand is disordered over three positions (with refined occupancies of 0.296, 0.406, and 0.296), with two of them being symmetrically related by a crystallographic 2-fold axis. Therefore the aforementioned half TBAPy^{4-} is represented in the ASU with three sets of chemically equivalent atoms, as presented in Supplementary Figure 2. Assuming that the formula unit comprises the content of the ASU (Z = 8, Z' = 0.5), the formula of **SION-19** is $Zn_{1.5}(TBAPy)_{0.5}(Ade)O_{0.25}$. Contribution of the disordered solvent molecules found in the structural voids of **SION-19** to the measured structure factors was quantified with the solvent masking procedure implemented in the Olex2 software.[6, 7] The unit cell was found to contain the total volume of 4393.6 Å^3 of structural voids carrying the electron count of 1217.8 e^{-}. Structural details and final refinement indicators are listed in Supplementary Table 1.

H-atom positions were derived from the molecular geometry (AFIX 43 for the aromatic H in TBAPy and Ade, AFIX 93 for the $–NH_2$ group of Ade), and refined using the riding model. No significant difference electron density was found in proximity of $–NH_2$ group prior to the H-atom assignment, undermining a potential refinement based on electron density maxima.

Supplementary Table 1: Crystal data and structure refinement for **SION-19 -** CCDC 1855564.

Identification code	SION-19
Empirical formula	$C_{29.8}H_{31}N_{6.6}O_{8.75}Zn_{1.5}$
Formula weight	719.67
Temperature/K	100.0
Crystal system	orthorhombic
Space group	Ccce
a/Å	10.7402(3)
b/Å	30.6236(7)
c/Å	42.6282(11)
α/°	90
β/°	90
γ/°	90
Volume/Å^3	14020.6(6)
Z	16
ρ_{calc}g/cm^3	1.364
μ/mm^{-1}	1.263
F(000)	5936.0
Crystal size/mm^3	0.09 × 0.09 × 0.03
Radiation	synchrotron (λ = 0.7519)
2Θ range for data collection/°	3.464 to 56.06
Index ranges	-11 ≤ h ≤ 11, -38 ≤ k ≤ 38, -53 ≤ l ≤ 53
Reflections collected	30744
Independent reflections	6454 [R_{int} = 0.0376, R_{sigma} = 0.0295]
Data/restraints/parameters	6454/554/425
Goodness-of-fit on F^2	1.136
Final R indexes [I>=2σ (I)]	R_1 = 0.1039, wR_2 = 0.2779
Final R indexes [all data]	R_1 = 0.1165, wR_2 = 0.2885
Largest diff. peak/hole / e Å^{-3}	0.91/-0.59

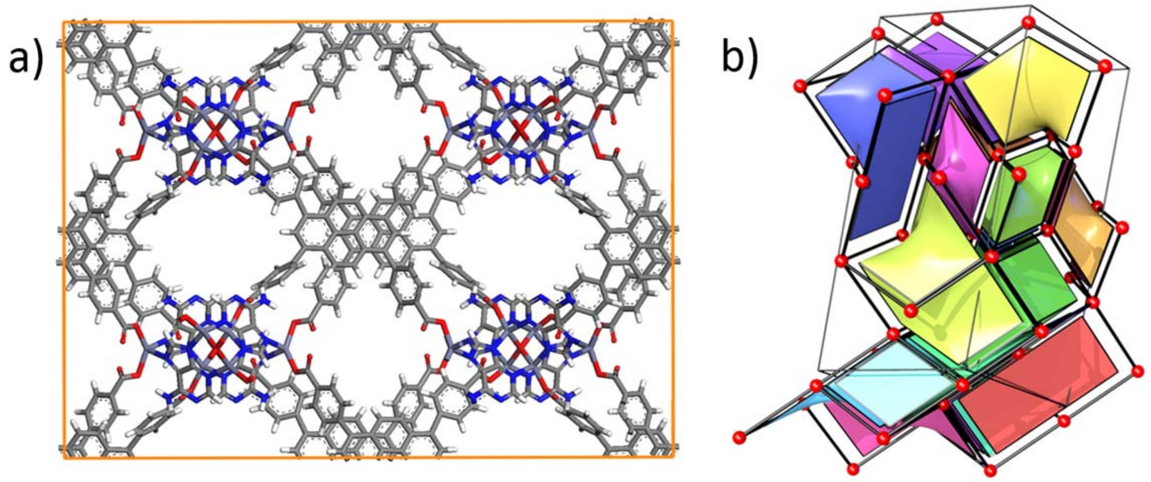

Supplementary Figure 3: Reducing the **SION-19** structure a) to the underlying 'kcs1' net, b) TOPOS 4.0 was used with the cluster simplification method to obtain the net in b).[8]

Supplementary Discussion 3. Scanning Electron Microscopy (SEM).

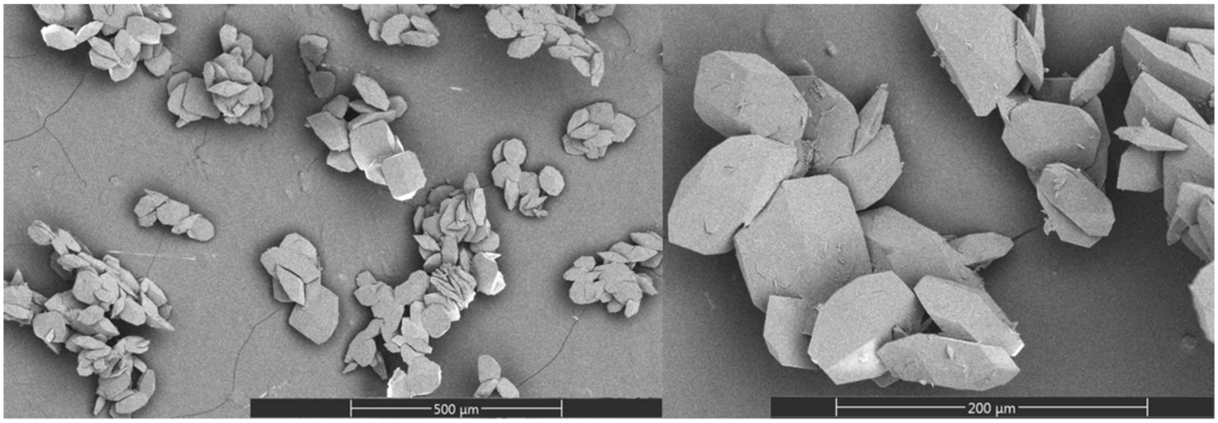

Supplementary Figure 4: SEM of **SION-19** crystals showing homogeneity, shape (truncated rhombic bipyramid like crystals), and size. Image on the left is at 500 µm, while the right is at 200 µm.

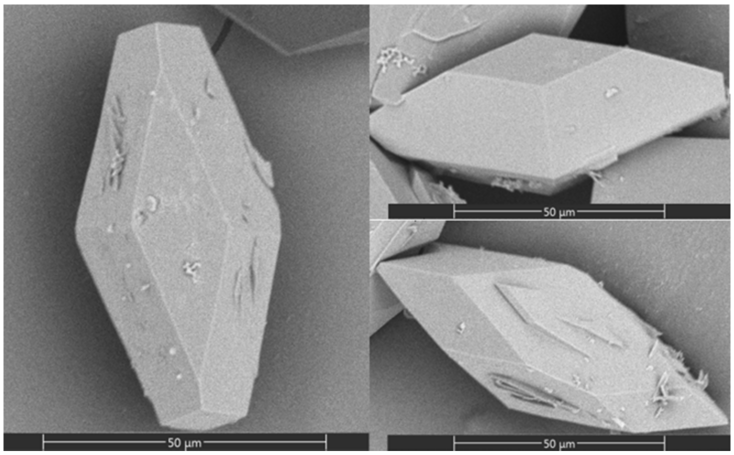

Supplementary Figure 5: SEM of **SION-19** crystals showing the truncated rhombic bipyramid like morphology. All images taken at 50 µm.

Supplementary Discussion 4. Solid State Characterization.

Supplementary Discussion 4.1. Characterization of SION-19.

The phase purity of **SION-19** was confirmed by the comparison of the experimental PXRD pattern with the simulated generated from the single crystal structure. **SION-19** was found to be air stable over a period of 6 months at ambient temperature while stored in a vial.

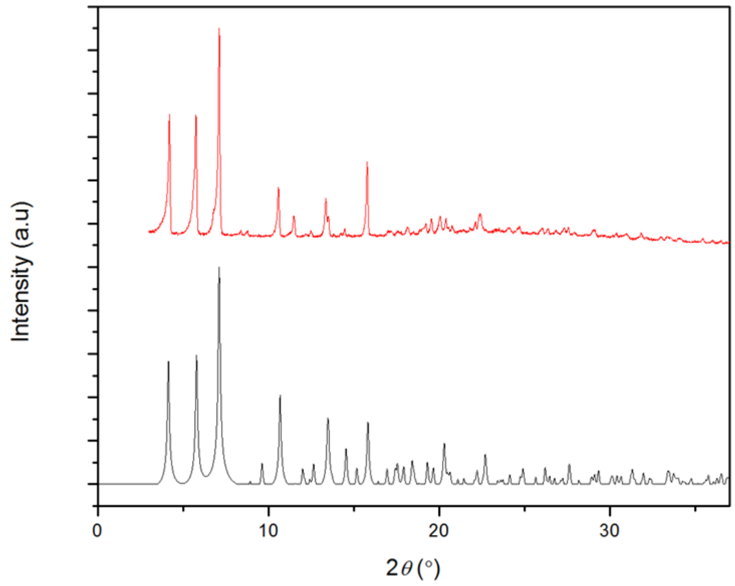

Supplementary Figure 6: PXRD of **SION-19**. Color scheme: black, theory; red, experimental.

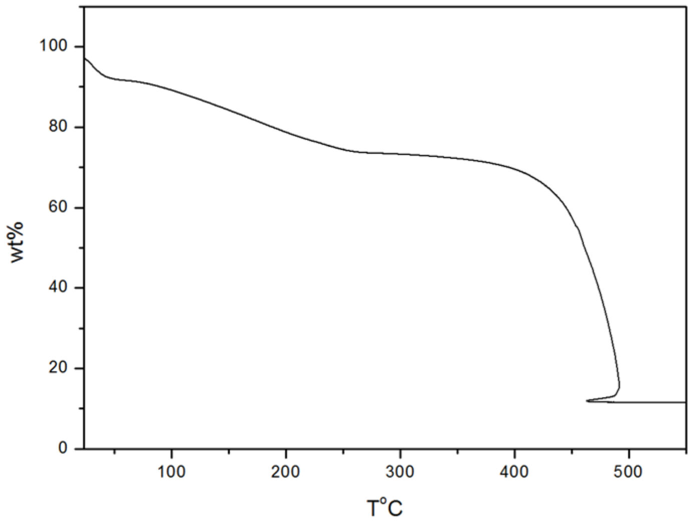

Supplementary Figure 7: TGA analysis of **SION-19.**

EA of the bulk phase reveals that the overall structure of **SION-19**, $[Zn_{1.5}O_{0.25}(Ade)(TBAPy)_{0.5}]$ $(NH_2Me_2)_{0.5}\cdot(DMF)_{1.7}\cdot(H_2O)_{4.0}$, is comprised of 26.9% guest molecules. This is in good agreement with the 27.9% loss in the TGA (Supplementary Figure 7). Here, the TGA profile shows two plateaus; the first occurs between 30-50˚C and has an 8.8% weight loss attributed to the removal of guest H_2O molecules (calculated 9.2% loss), while the second occurs at 50-380 ˚C and corresponds to DMF and $Me_2NH_2^+$. Following the loss of the cations, the framework begins to decompose.

Supplementary Discussion 4.2. Characterization of SION-19'.

The phase purity of **SION-19'** was confirmed by the comparison of the experimental PXRD pattern with the simulated generated from the single crystal structure from the as made **SION-19** material. **SION-19'** was found to be air stable over a period of 6 months at ambient temperature while stored in a vial.

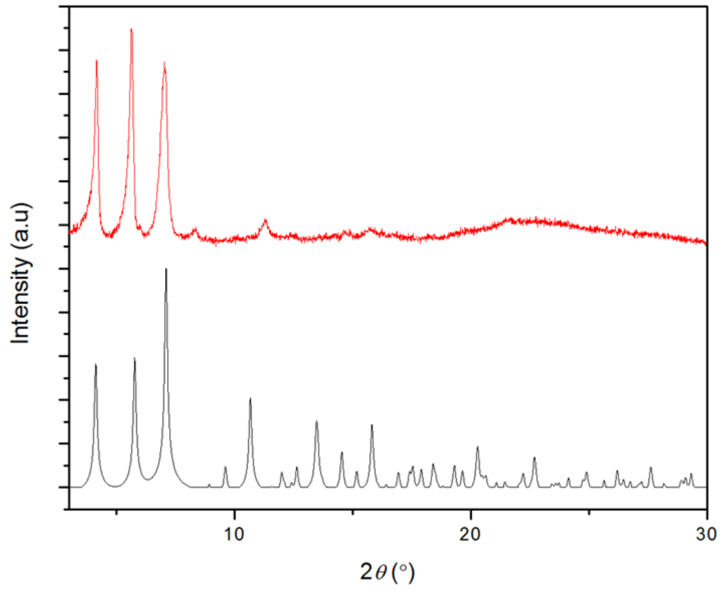

Supplementary Figure 8: PXRD of **SION-19'**. Color scheme: black, theory; red, experimental.

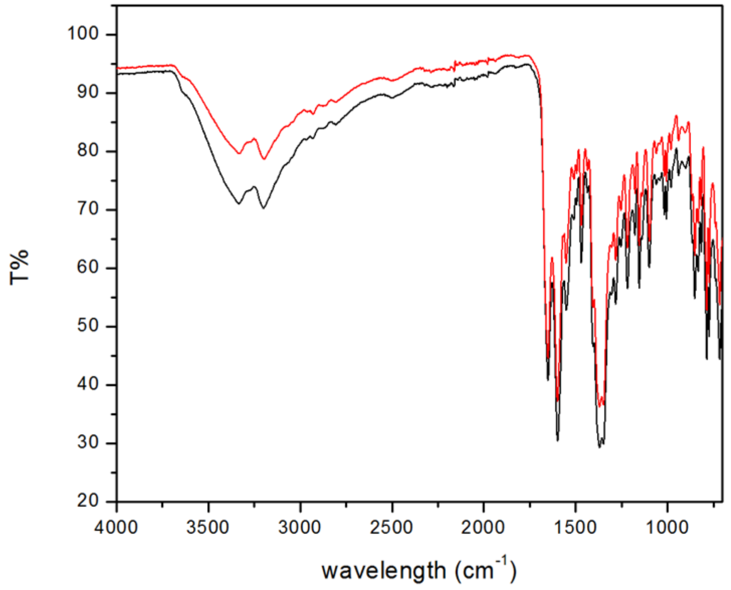

Supplementary Figure 9: IR. Colour scheme: Black, **SION-19**; red, **SION-19'**.

Supplementary Table 2: Elemental Analysis of **SION-19'**.

	**Anal. Cald for:**	**Theory**	**Experimental**
**SION-19'**	$[Zn_{1.5}O_{0.25}(Ade)(TBAPy)_{0.5}]$ $(NH_2Me_2)_{0.5}\cdot(H_2O)_{0.2}$	C 55.99<br>H 3.22<br>N 12.83	C 55.78<br>H 3.13<br>N 13.19

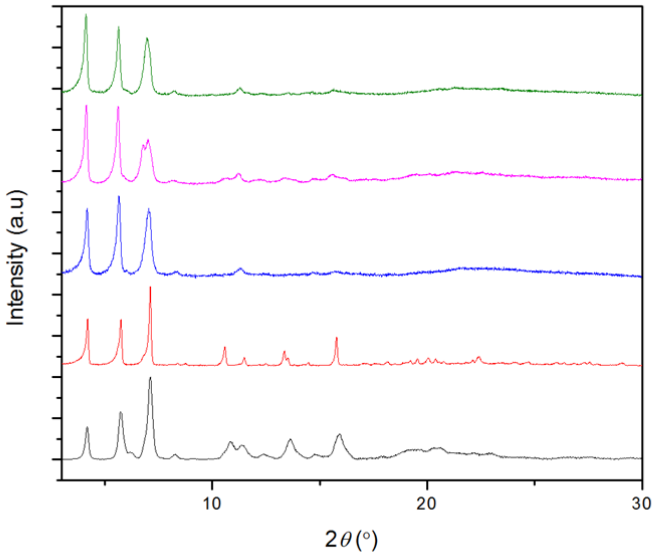

Supplementary Figure 10: Stability of **SION-19'** in DMF and EtOH. Color scheme: black, theory; red, **SION-19**; blue, **SION-19'**; pink, **SION-19'** immersion in DMF for 24 hrs; green, **SION-19'** immersion in EtOH for 24 hrs.

Supplementary Discussion 4.3. Le Bail Fit of SION-19'.

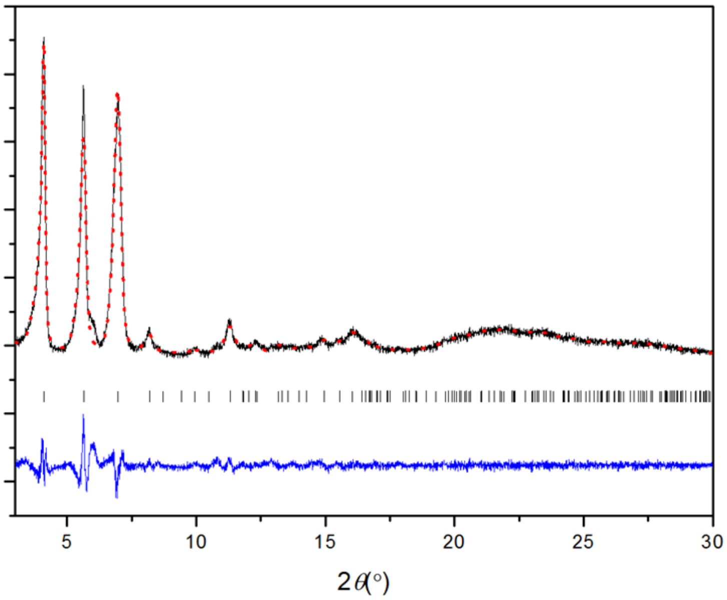

Supplementary Figure 11: Le Bail fit. Final observed (solid lines), calculated (circles) and difference (below) XRD for the Le Bail fit refinements of **SION-19'**.

Supplementary Discussion 5. XPS of SION-19' Compared to H9Ade.

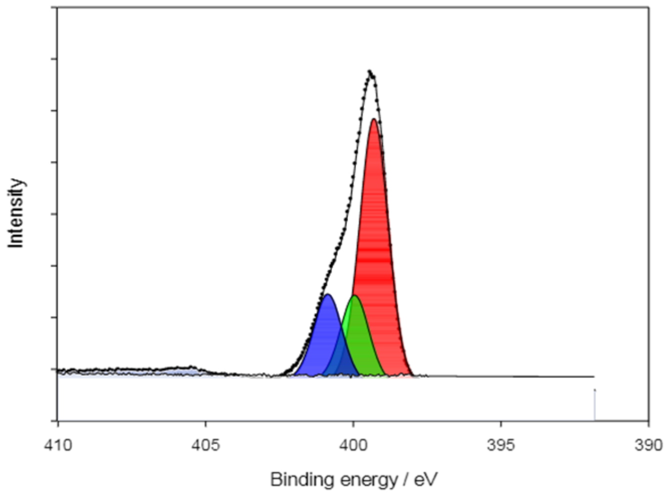

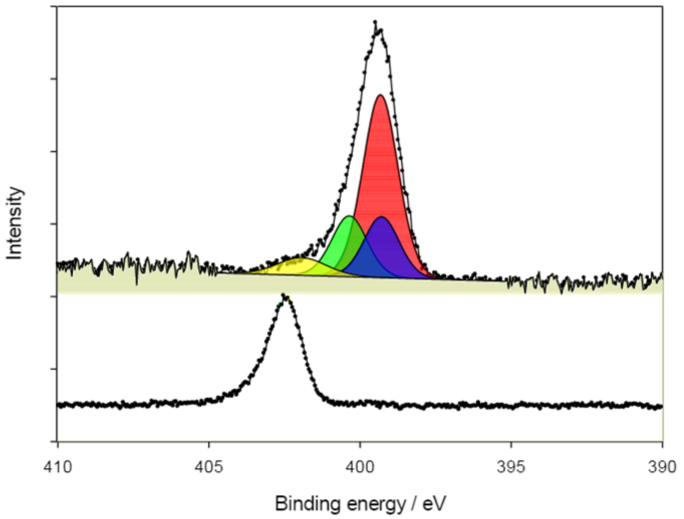

Supplementary Figure 12: Top: XPS N1s region of solid state Ade; Right, top: XPS N1s region of **SION-19**; Bottom, bottom: N1s XPS of NH_2Me_2Cl. All fitting parameters are described in the text below.

Three Gaussian–Lorentzian peaks were used to fit the spectrum, corresponding to the three peaks observed in the gas phase. Component A (red) corresponds to N1, N3, N7 of free adenine (H9Ade), component B (green) corresponds to N6 and component C (blue) corresponds to N9 (Supplementary Figure 12). The component area ratio A : B : C was constrained according to the number of N atoms it comprised, i.e. to the ratio 3 : 1 : 1. The full width at half maximum (FWHM) of all components were constrained to be equal to give a more physically realistic model. The resulting components A, B and C have binding energies of 399.30, 399.96, and 400.87 eV respectively. The binding energy difference between components A and B and A and C is 0.66 eV and 1.57 eV respectively, compared with 1.3 eV and 2.3 eV in the gas phase molecule measured by Plekan et al.[9] Thus it can be seen that the N1s binding energies from the different N atoms in Ade become closer together in the solid state, presumably due to the influence of intermolecular

interactions, especially hydrogen bonding. The model applied to solid state H9Ade was used to model spectra taken from **SION-19**. The binding energies of components A, B and C in **SION-19** are found at 399.33, 400.46 and 399.33 eV respectively (Supplementary Table 4). Additionally, a fourth component was observed at 402.2 eV. This feature matches closely to the N1s peak observed in dimethyl ammonium chloride (402.1 eV), suggesting that this or a similar ammonium ion is present within **SION-19**. This observation confirms the presence of N^+ cations within the channels.

Supplementary Table 3: Binding energies of the fitted components for the N1s spectra. Compounds A, B and C were constrained to have the same FWHM, and the peak areas were constrained to a ration of A:B:C of 3:1:1. $^{\alpha}$Values in brackets are the differences of binding energies from component A.

	**Binding Energy (eV) of Component**			
	**A**	**B**	**C**	**D**
**9HAde**	399.30	399.96 (+0.66)$^{\alpha}$	400.87 (1.57)$^{\alpha}$	---
**SION-19**	399.33	400.46 (-0.1.13)$^{\alpha}$	399.33 (0)$^{\alpha}$	402.2
**$NH_2Me_2^+$**	---	---	---	402.1

Supplementary Discussion 6. Porosity.

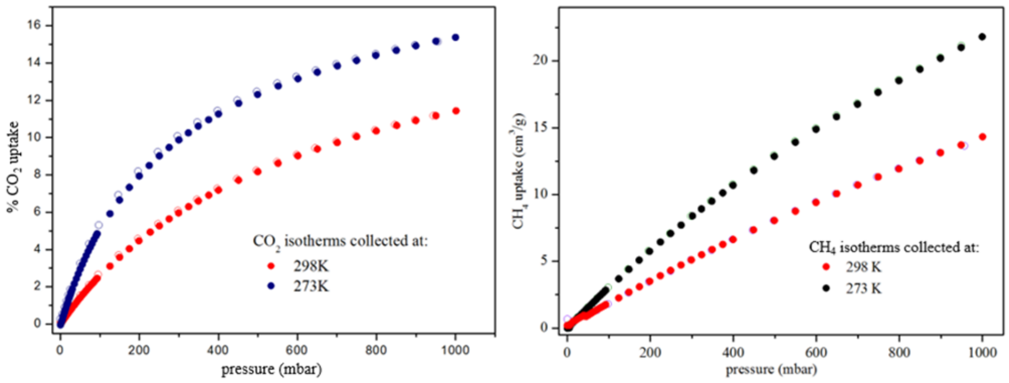

Supplementary Figure 13: CO_2 (left) and CH_4 (right) isotherms collected on **SION-19'** at 298 and 273 K.

Supplementary Discussion 7. Thy loading of SION-19'.

Supplementary Discussion 7.1. Computational Methods.

Due to the disorder of the pyrene rings in **SION-19**, the structure was initially prepared for computational simulations by removing all pyrene groups except for one. This was to ensure equidistance of the pyrene rings in the *a*-direction of the lattice (~5.5 Å). It was assumed that the disorder in these ligands is due to conformational flexibility (this was observed in the MD simulations discussed below), and thus the choice of which TBAPy ligand to keep was somewhat arbitrary. All additional disorder related to the positions of the ligand's benzoic acid groups could be eliminated once the choice of pyrene ring was made.

All DFT calculations were performed with the Vienna Ab-initio Simulation Package (VASP) version 5.3.5 using the PBE exchange-correlation functional and the Projector Augmented Wave method.[10-16] The Kohn-Sham orbitals were expanded in a plane-wave basis up to a 1000 eV cutoff and the Brillouin zone was sampled using a 2x1x1 k-point grid centered at the gamma point.

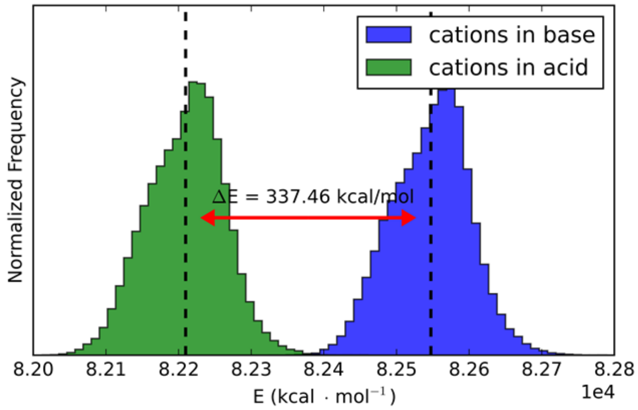

Supplementary Figure 14: Histogram of average internal energy of the **SION-19'** system containing charge-balancing $[NH_2Me_2]^+$ ions in the acid (green) and base (blue) pores. The total average energy difference is 337.46 kcal/mol in favor of the acid pore. There are 24 cations in the supercell of the simulation, resulting in ΔE = 14 kcal/mol per cation in favor of the acid pore. The simulations were run in the NPT ensemble, with energy data collected every femtosecond over the span of 2 nanoseconds.

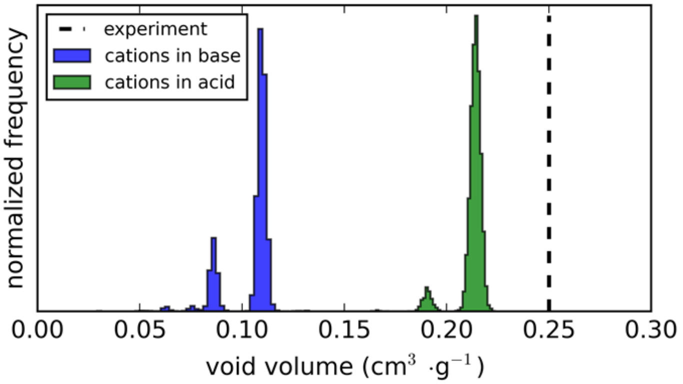

Supplementary Figure 15: Histogram of the accessible volumes of **SION-19'** computed over a 2ns NPT MD simulation. Snapshots of the structure were taken every 2 ps. The x-axis represents the probe occupiable accessible volume, computed using the technique presented in Ongari et al. as implemented in the zeo++ code version 0.3.[17] The average pore volume is computed to be: base (blue) = 0.104 cm^3/g, acid (green) = 0.213 cm^3/g. The experimental value of 0.246 cm^3/g is in agreement with the accessible volume computed when cations are in the acid pore.

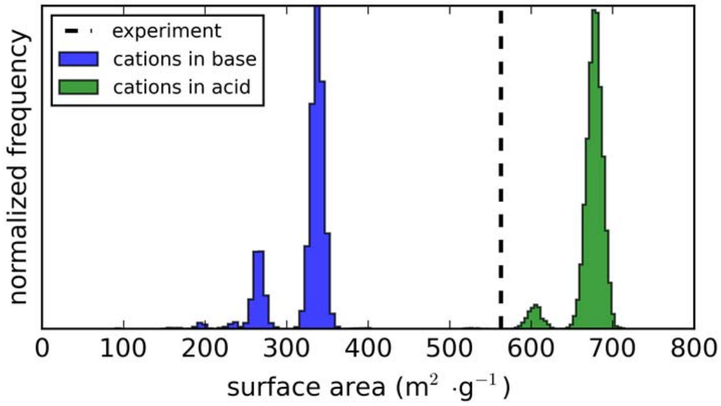

Supplementary Figure 16: Histogram of the surface area of **SION-19'** computed over a 2ns NPT MD simulation. Snapshots of the structure were taken every 2 ps. The x-axis represents the geometric surface area using a spherical probe of 1.82 Å using the zeo++ code version 0.3.[17] The average surface area is computed to be: base (blue) = 321 m^2/g, acid (green) = 672 m^2/g. The experimental value of 563 m^2/g is in better agreement with the surface area computed when cations are in the acid pore.

The unit-cell of **SION-19** has a net anionic charge of -8 without charge-compensating extra-framework cations. Partial atomic charges for this MOF were assigned by computing the DFT electrostatic potential, ensuring an additional 8 electrons were included in the MOF (setting NELECT=3072 in VASP), and solving the RESP-like equations as implemented in the REPEAT method, while constraining the system total charge to -8.[18] A similar procedure was performed for a lone dimethyl ammonium cation *in vacuo* (the extra-framework cation in this study), while constraining the total charge to +1 in both its REPEAT and VASP

calculations (setting NELECT=20 in the latter). In all subsequent classical simulations, 8 dimethyl ammonium cations with the partial charges computed in the procedure above were placed in the simulation unit cell to balance the net charge. The atomic charge assignments and Lennard-Jones parameters for the dimethyl ammonium atoms are presented in Supplementary Table 4, with atomic labels shown in Supplementary Figure 18.

Molecular Dynamics simulations (MD) were performed in the Large-scale Atomic/Molecular Massively Parallel Simulator (LAMMPS) version 10Aug15.[19] DREIDING potentials were used to model the fully-flexible MOF, cations, and free thymine nucleobases in the pores.[20] Included with the typical non-bonded interactions of DREIDING is a 3-bodied Morse hydrogen bond potential described in equation SE1.

$$E_{Morse}^{H-bond} = D_0\left(e^{-2\alpha(r-R_0)} - 2e^{-\alpha(r-R_0)}\right)cos^n\theta \quad \text{(SE1)}$$

Where D_0 is the well-depth of the potential, α is an exponential factor, and R_0 is the equilibrium H-bond distance between the H-bond donor and acceptor atoms, while θ describes the angle formed between the heavy atoms and the hydrogen. In this study, the value for n was set to 2. The original DREIDING parameters for this potential were modified by Liu *et al.* to improve energetics of hydrogen bonds in nitrogen-containing dendrimers.[21] This work uses the modified parameters, where possible, of Liu *et al.* to model the H-bonds of Ade-Thy. Exceptionally, we have adjusted the N_R – N_R hydrogen bonding equilibrium distance (R_0) to match that of the DFT optimized distance of 2.72 Å. The values of the H-bonding parameters used in this work are presented in Supplementary Table 5.

No partial atomic charges were assigned to Thy, due to observed over-binding of Thy to Ade in isolated simulations. The energy of the base-pair was computed to be -19.6 kcal/mol with the 3-bodied Morse potentials. This is comparable to the DFT binding energy of -18.24 kcal/mol.

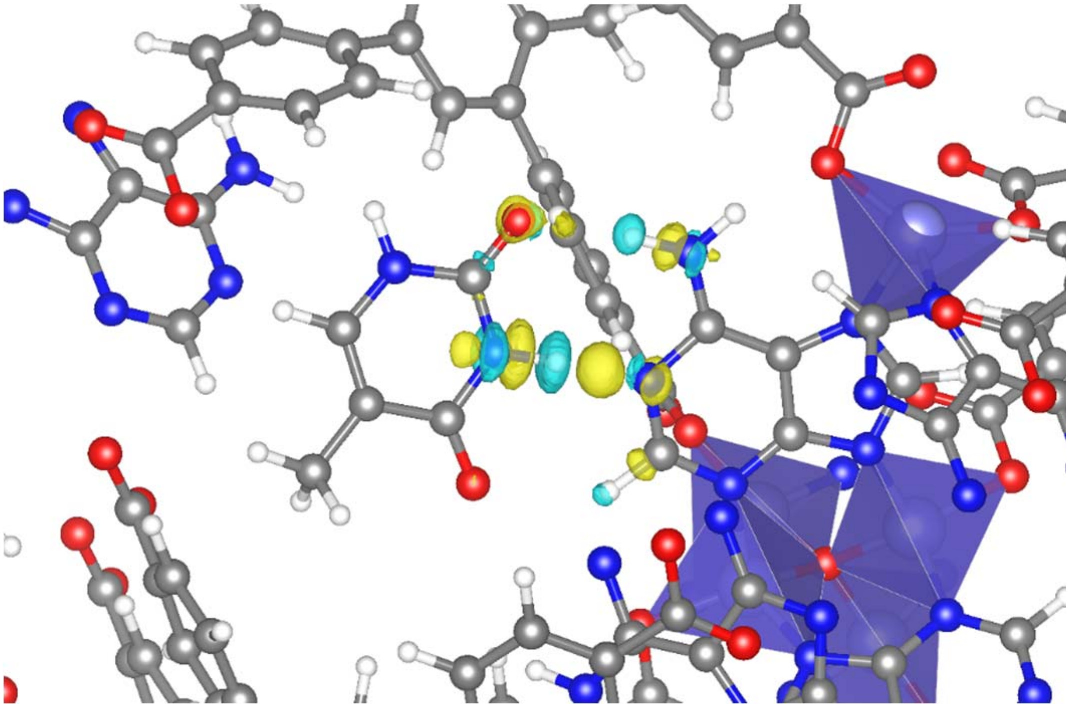

Supplementary Figure 17: Charge difference isosurface between hydrogen bonded Thy in **SION-19'** and their isolated species. The isovalue of this plot is set to 0.004 e/Å^3. Negative isosurfaces presented in light blue and positive isosurfaces shown in yellow. Bader population analysis on the charge density of these species suggest a polarization of charge in the hydrogen bonded structure. The Thy oxygen becoming slightly positive (+0.06e) and the Thy hydrogen becoming slightly negative (-0.06e).

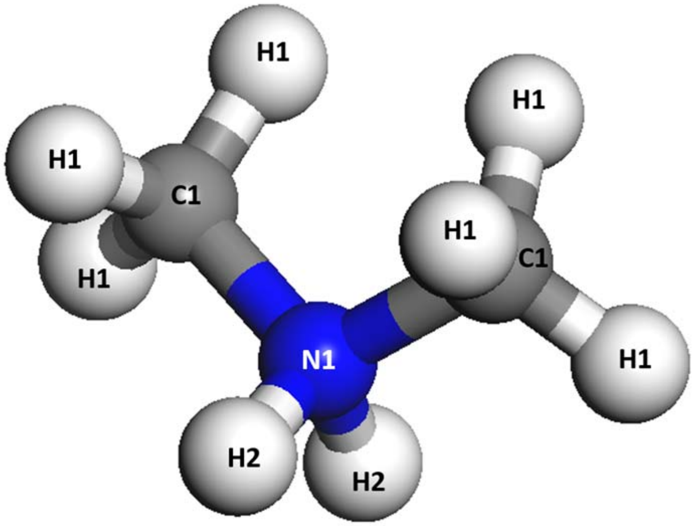

Supplementary Figure 18: Dimethyl ammonium cation with atomic labels.

Supplementary Table 4: non-bonded interaction parameters of dimethyl ammonium cation. Atom labels correspond to the labels presented in Supplementary Figure 18.

Atom	Parital charge (e)	Lennard-Jones ε (kcal/mol)	L-J σ (Å)
N1	-0.12	0.0774	3.263
H1	+0.33	0.0152	2.846
H2	+0.17	0.0152	2.846
C1	-0.28	0.0951	3.473

Supplementary Table 5: Morse H-bond potentials used in this work for framework Ade and free Thy.

Donor - Acceptor	D_0 (kcal/mol)	α (1/Å)	R_0 (Å)
N_R – N_R	9.50	1.838	2.72
N_R – N_3	8.45	1.761	2.84
N_R – O_2	9.50	1.818	2.75
N_R – O_R	9.50	1.667	3.00

Simulations sampling the canonical ensemble were performed for a total of 2.4 ns (2 ns production and 400 ps equilibration). During equilibration, the framework, cations and Thy molecules were subjected to three separate Langevin thermostats to reduce the undesirable oscillations observed with Nosé-Hoover thermostats, while the production run was governed by individual Nosé-Hoover thermostats with 3 chains each.

The zeo++-0.3 program was used to compute the time dependent surface area of **SION-19**,[17, 22-24] where every 100 fs, a snapshot of the simulation was extracted using the MDAnalysis python library.[25, 26] For each snapshot, a probe radius of 1.82 Å was used to mimic a N_2 BET-like surface area, and 10,000 points were sampled on the MOF surface.

Supplementary Discussion 7.2. Characterization of SION-19@Thy.

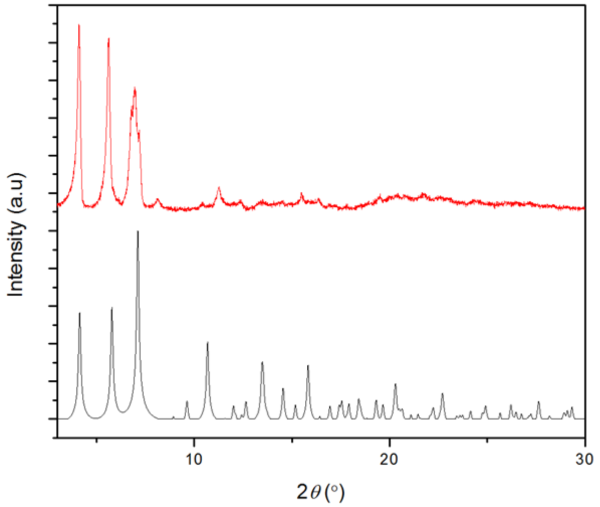

Supplementary Figure 19: PXRD of **SION-19@Thy**. Color scheme: black, theory; red, experimental.

Supplementary Table 6: Elemental Analysis of **SION-19**

	**Anal. Cald for:**	**Theory**	**Experimental**
**SION-19**	$[Zn_{1.5}O_{0.25}(Ade)(TBAPy)_{0.5}]\cdot(NH_2Me_2)_{0.5}$ $(Thy)_{0.95}(EtOH)_{1.1}(H_2O)_{1.5}$	C 52.86<br>H 4.32<br>N 13.05	C 53.17<br>H 4.13<br>N 13.32

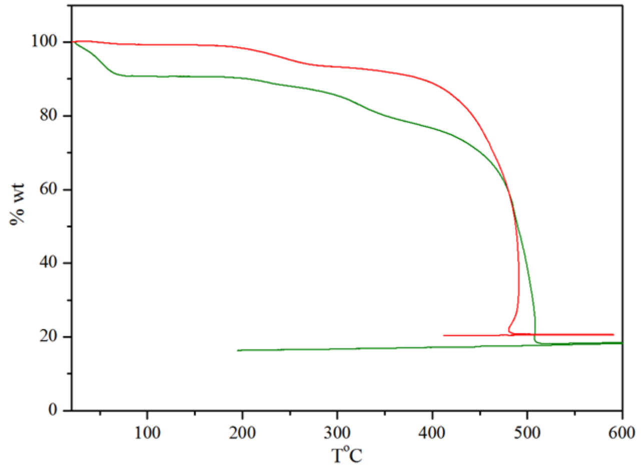

Supplementary Figure 20: TGA analysis**.** Color scheme: red, **SION-19'**; green, **SION-19@Thy**

The TGA for **SION-19'** and **SION-19@Thy** (Supplementary Figure 20) were collected up to 600 ˚C under air. For **SION-19'** there is a minor loss at 170 ˚C of 0.2 % corresponding to the removal of H_2O absorbed upon exposure to air. The loss observed between 170-355 ˚C is attributed to the removal of $[NH_2Me_2]^+$ followed

by the collapse of the material. For **SION-19@Thy**, the first plateau corresponds to a loss of EtOH and H_2O, while a 16.2 % loss between 170-400 ˚C is synonymous with the loss of cations, Thy molecules, and subsequent collapse of the MOF.

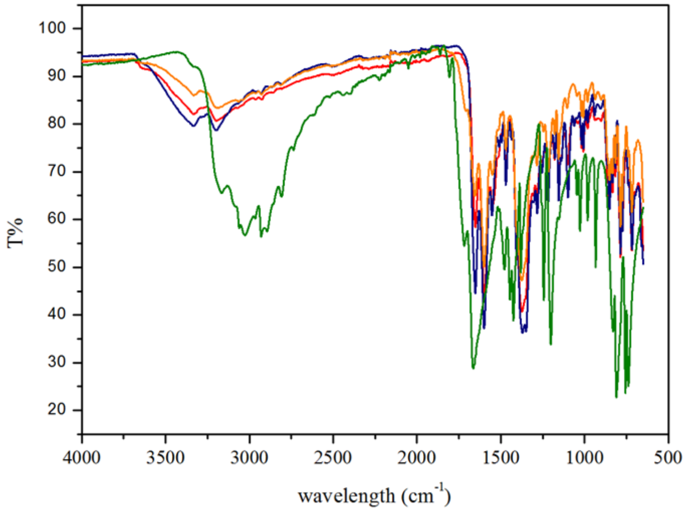

Supplementary Figure 21: IR. Colour scheme: Blue, **SION-19**; red, **SION-19'**; orange, **SION-19@Thy**; green, Thy.

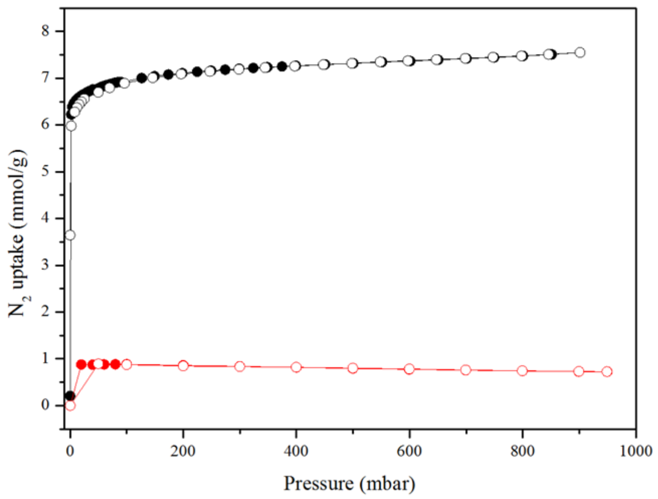

Supplementary Figure 22: Type I N_2 isotherm measured on **SION-19'** (black) and **SION-19@Thy** (red) at 77 K and 1 bar (filled symbols: adsorption, empty: desorption).

Supplementary Discussion 7.3. MAS ^1H and ^{13}C NMR Experiments.

Variable-temperature ^{13}C (125.7 MHz) NMR spectra were acquired on a Bruker Advance III 11.7 T spectrometer equipped with a 3.2 mm low-temperature CPMAS probe. A recycle delay of 2 to 4 seconds, and a contact time of 3 ms were used in the CP experiment. The spectra were referenced to the CH signal of solid adamantane (29.06 ppm), used as a secondary reference.

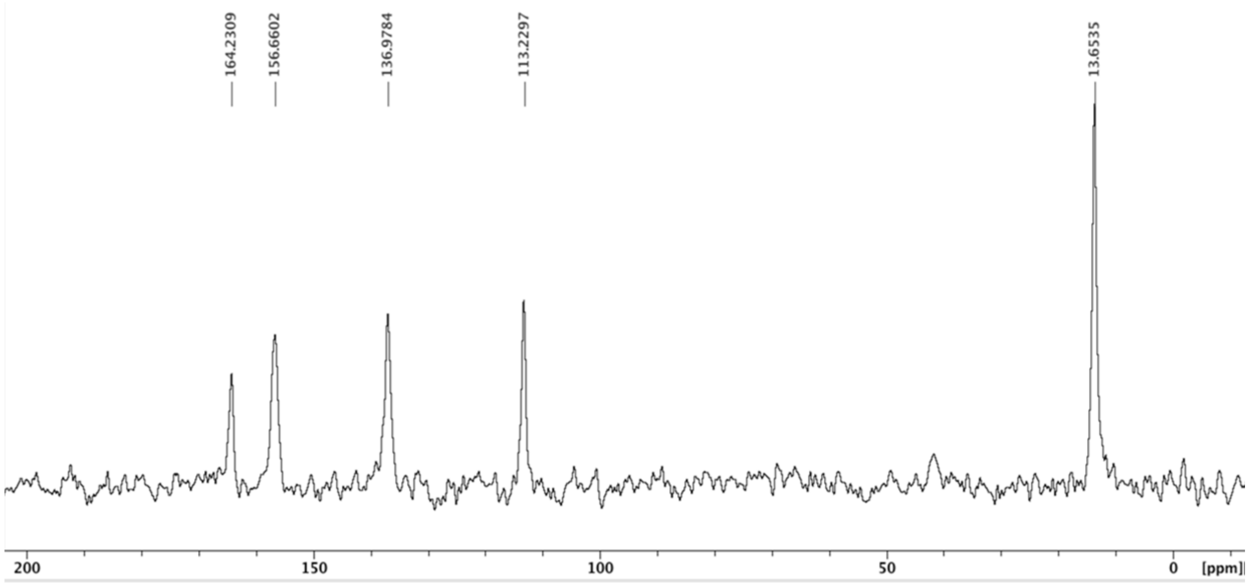

Supplementary Figure 23: MAS ^1H NMR of Thymine, 300K, 12000 Hz.

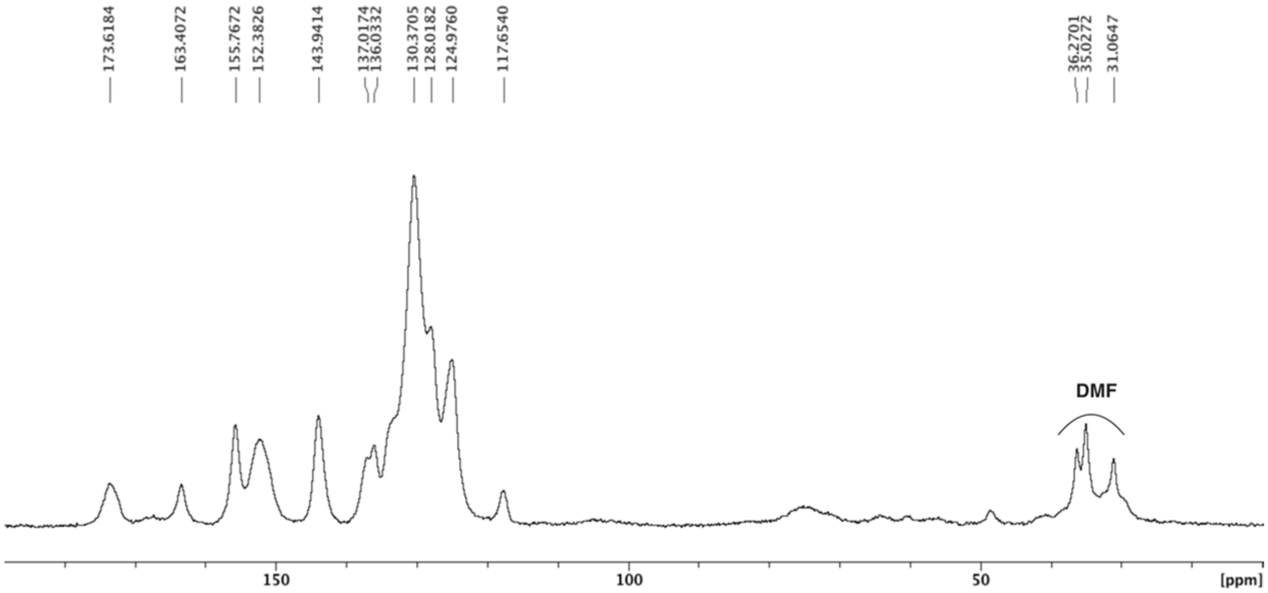

Supplementary Figure 24: MAS ^{13}C NMR of **SION-19**, 300K, 12000 Hz.

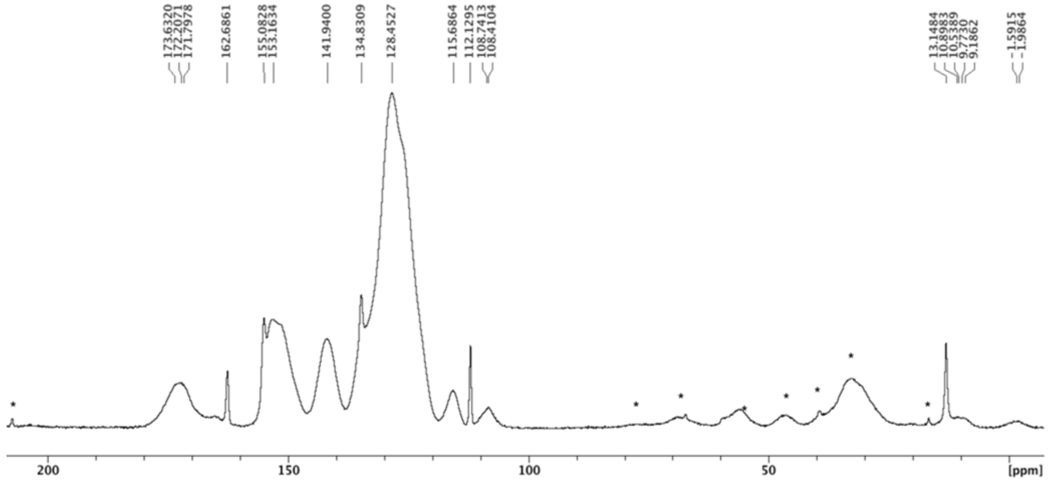

Supplementary Figure 25: MAS ^{1}H-^{13}C NMR of **SION-19@Thy**, 103K, 12000 Hz.

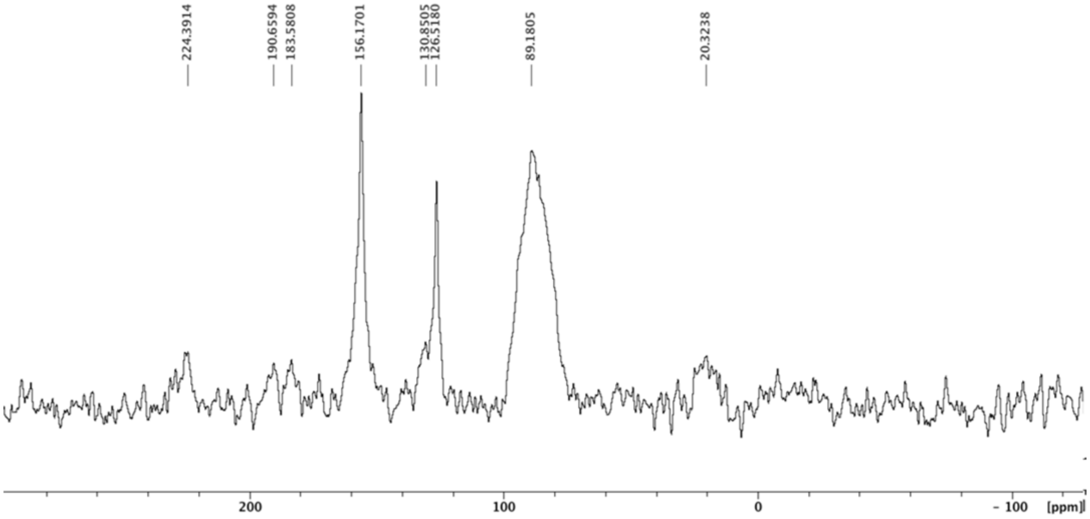

Supplementary Figure 26: MAS ^{1}N CP NMR of **SION-19@Thy**, 100K, 12000 Hz. Peaks correspond to: δ Thy (N3), 156.1701; Thy (N1) 126.5180-130.8505; and Ade (NH_2) 89.1805.

Supplementary Discussion 8. Characterization of Thymine Dimer and SION-19'@UV-Thy.

Supplementary Discussion 8.1. Synthesis and Characterization of Thy<>Thy.

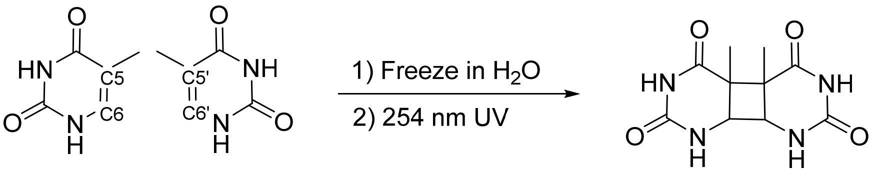

Supplementary Scheme 1: Synthesis of cyclobutane thymine photodimers and possible radical reaction mechanism.

The synthesis of the cyclobutane Thy photodimers was adapted from previously reported methods (Supplementary Scheme 1).[27-29] In a 1 L volumetric flask, Thy (360 mg, 2.85 mmol) was dissolved in Millipore water. The solution was transferred into a plastic container, which was placed in a Styrofoam container filled with dry ice, and rapidly frozen. The solid block of ice was broken up, placed under a UV-lamp, and irradiated at 254 nm for 5 hours. This procedure was repeated twice to ensure a high yield of Thy<>Thy. After, the water was removed by rotary evaporator, and washed with hot absolute ethanol to remove any unreacted Thy. Yield 6 % (47 mg, 0.183 mmol), ^{1}H NMR (400 MHz, DMSO): δ 7.63 (s, 2H); 3.68 (s, 2H); 1.31 (s, 6H).[30]

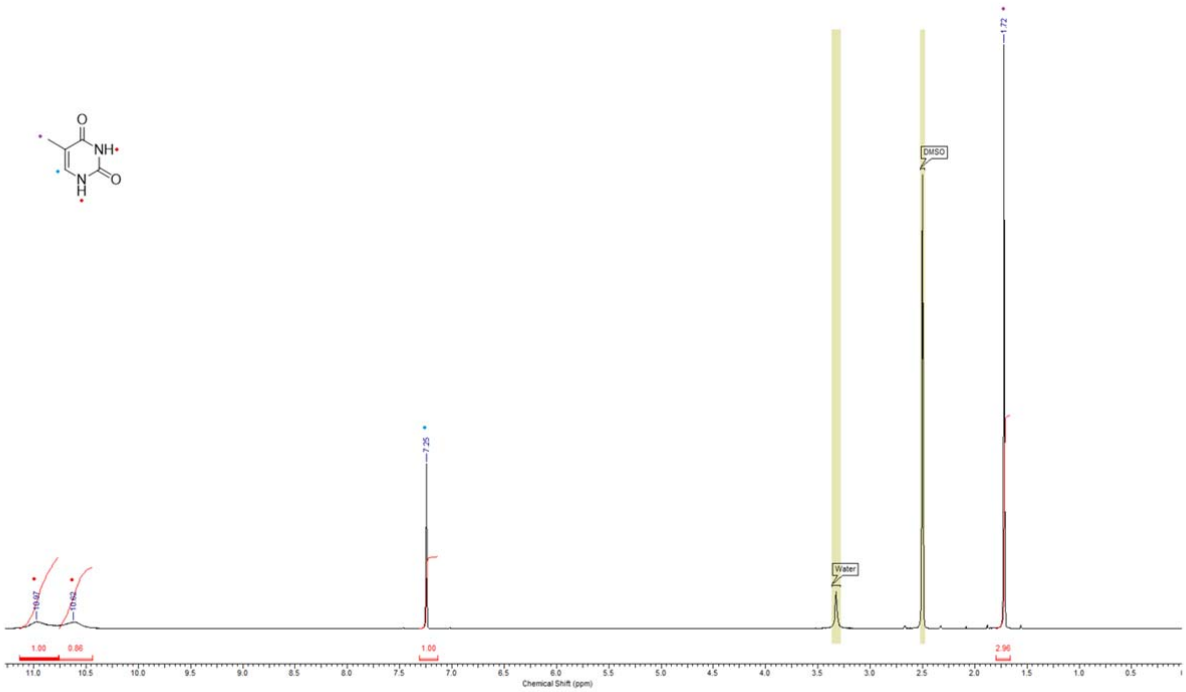

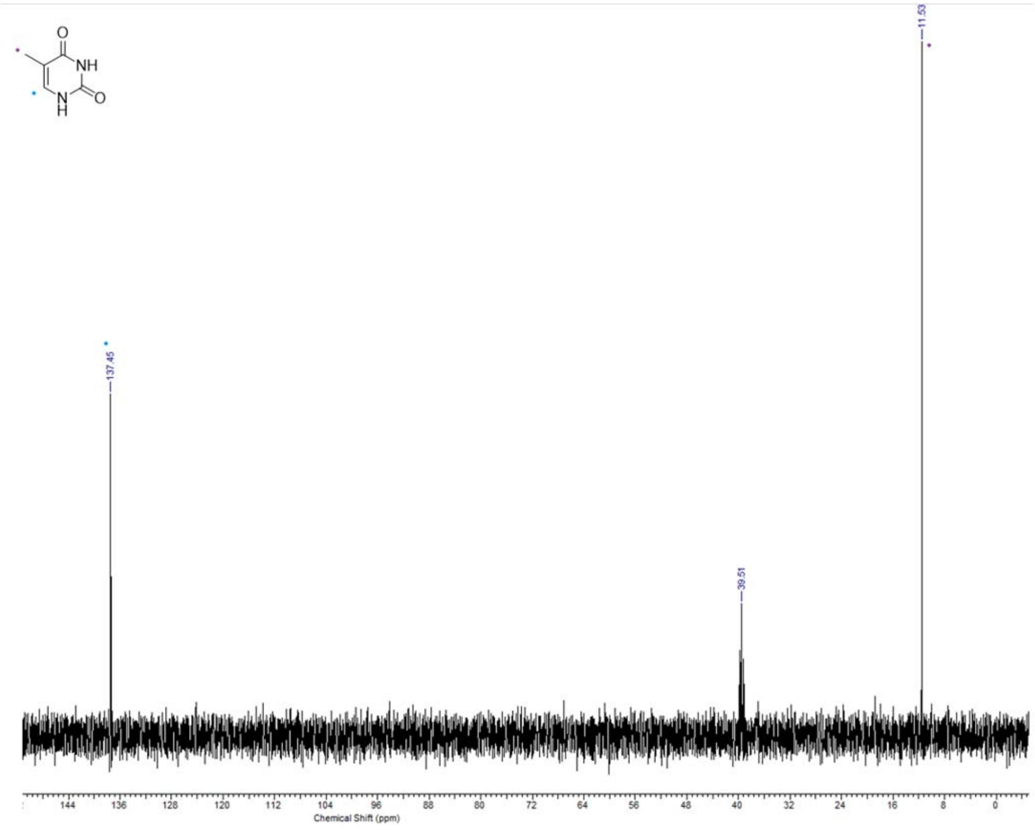

Supplementary Figure 27: Top: ^{1}H NMR and bottom: DEPT-135 ^{13}C of Thy exposed for 72 hours of UV light (254 nm). Measurements done in $_{d}$-DMSO.

Supplementary Discussion 8.2. Loading SION-19' with Thy<>Thy.

Following the same procedure previously discussed for the Thy isotherms, desolvated ground host material (50 mg), **SION-19'**, was immersed in MeCN (due to insolubility of Thy<>Thy in EtOH) which contained a 100% loadings of Thy<>Thy. Equilibrium was established after 24 hours, after which the material was filtered and allowed to air dry before routine characterization.

Supplementary Discussion 8.3. Characterization of SION-19@Thy<>Thy.

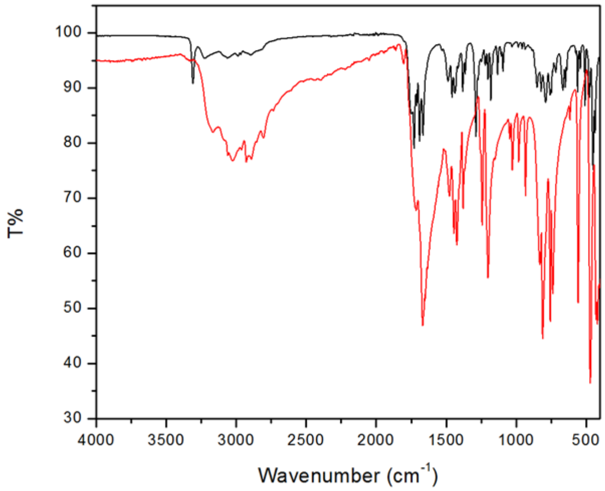

Supplementary Figure 28: IR spectrum. Color scheme: red, Thy; black, Thy<>Thy.

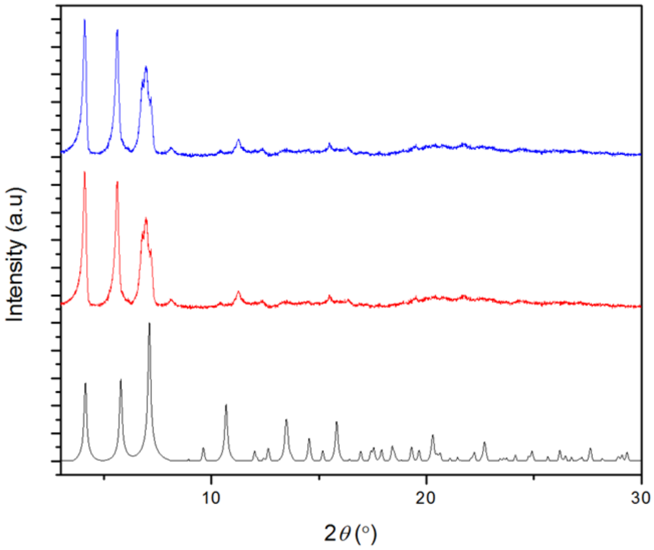

Supplementary Figure 29: PXRD. Color scheme: black, experimental; red, **SION-19@Thy(40%)**; blue, **SION-19@UV-Thy(40%)** exposed to UV/Vis for 24 hrs.

Supplementary Discussion 8.4. Ultra High Performance Liquid Chromatograph Mass Spectrometry.

Analysis of each sample by UHPLC-EIS/MS was performed at room temperature by taking 100 µL of each sample prepared in section 8.4 and diluting it with 1 mL of deionized water. Injection volumes of 30 µL were used along with the following solvent (HPLC grade Acetonitrile) gradient (Supplementary Table 7-8), and parameters for the Full MS-SIM and PRM scans.

Supplementary Table 7: The following solvent gradient for UHPLC-EIS/MS was applied to Thy, Thy<>Thy and destroyed material **SION-19@UV-Thy (20-80%)** that was exposed to 24 hrs of 254 nm UV.

	**Retention (min)**	**Flow (mL/min)**	**% MeCN**
**1**	0.000	0.300	2.0
**2**	0.000	0.300	2.0
**3**	1.000	0.300	2.0
**4**	5.000	0.300	20.0
**5**	6.000	0.300	80.0
**6**	7.000	0.300	80.0
**7**	7.100	0.300	2.0
**8**	10.000	0.300	2.0

Supplementary Table 8: Parameters and properties of the global settings, Full MS-SIM and PRM scans.

	**Properties of Methods**		
	**Global Settings**	**Full MS-SIM**	**PRM**
**Runtime (min)**	10	0 to 2.29<br>3.5-10	2.3-3.5
**Polarity**	-----	Positive	Positive
**Resolution**	-----	30,000	30,000
**AGC Target**	-----	1e6	5e5
**Max IT**	-----	200 ms	100 ms
**Scan Range**	-----	100-800 m/z	-----
**Isolation window**	-----	-----	1.0 m/z

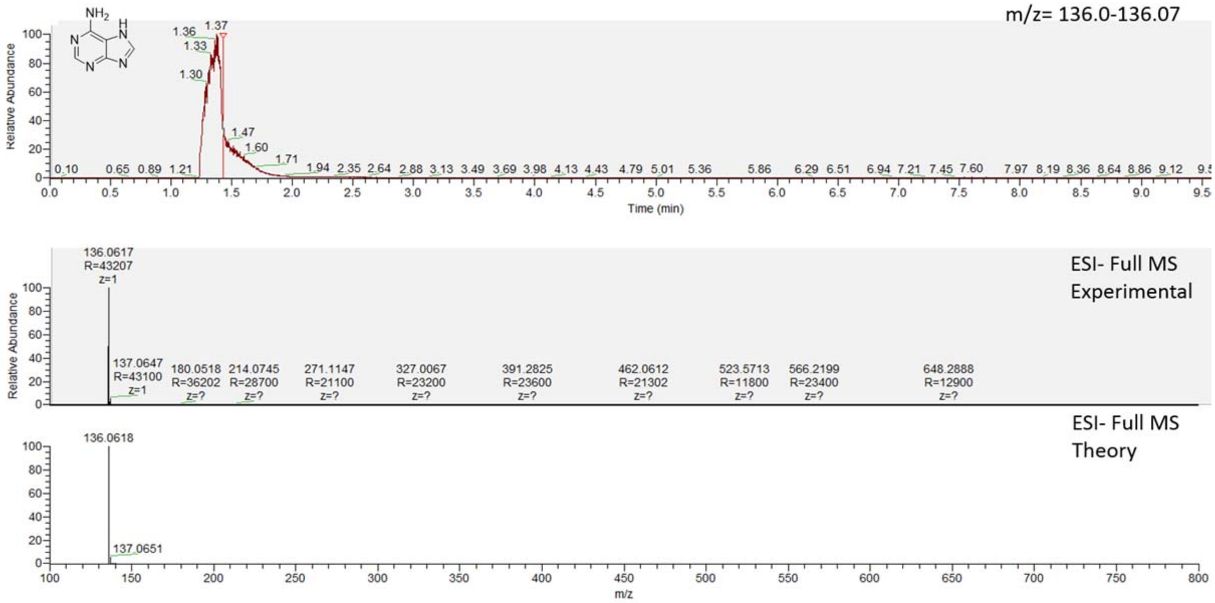

Supplementary Figure 30: Ade UHPLC-EIS/MS elution time (1.36 min), as well as the experimental and theoretical ESI-Full MS spectra. The parent ion peak here is found at 136.0617 m/z and is in good agreement with the theoretical ESI-Full MS peak at 136.0618 m/z. Here, a 250 nM sample was used along with 20 µL injection volumes.

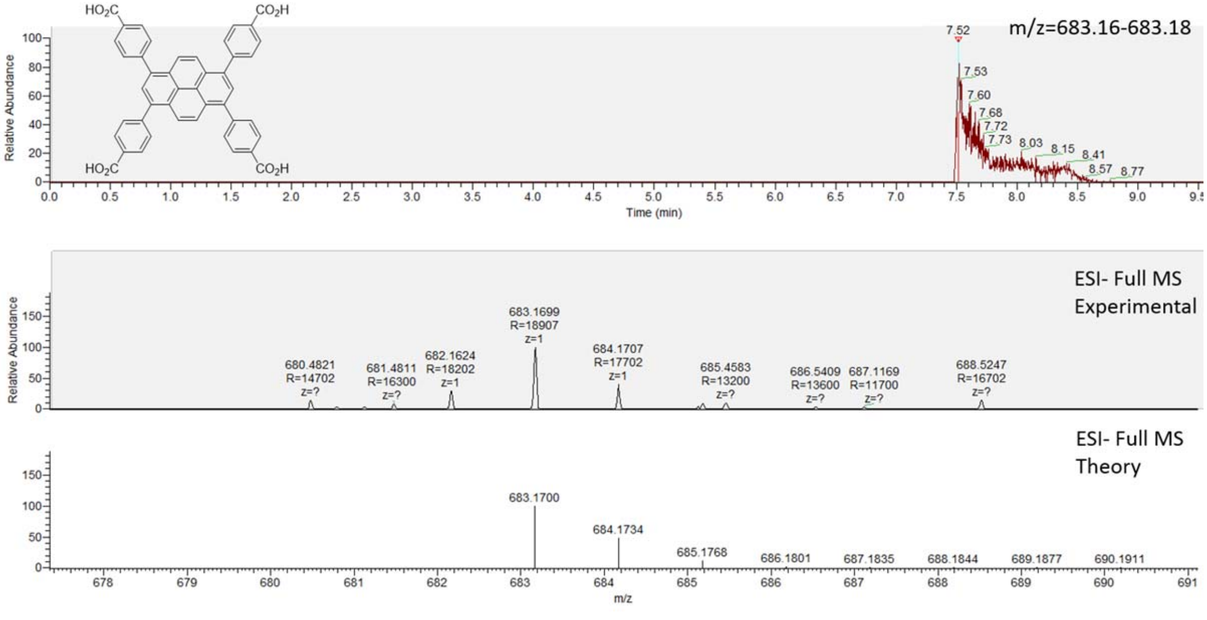

Supplementary Figure 31: TBAPy UHPLC-EIS/MS elution time (7.52 min), as well as the experimental and theoretical ESI-Full MS spectra. The parent ion peak here is found at 683.1699 m/z and is in good agreement with the theoretical ESI-Full MS peak at 683.1700 m/z. Here, a 250 nM sample was used along with 20 µL injection volumes.

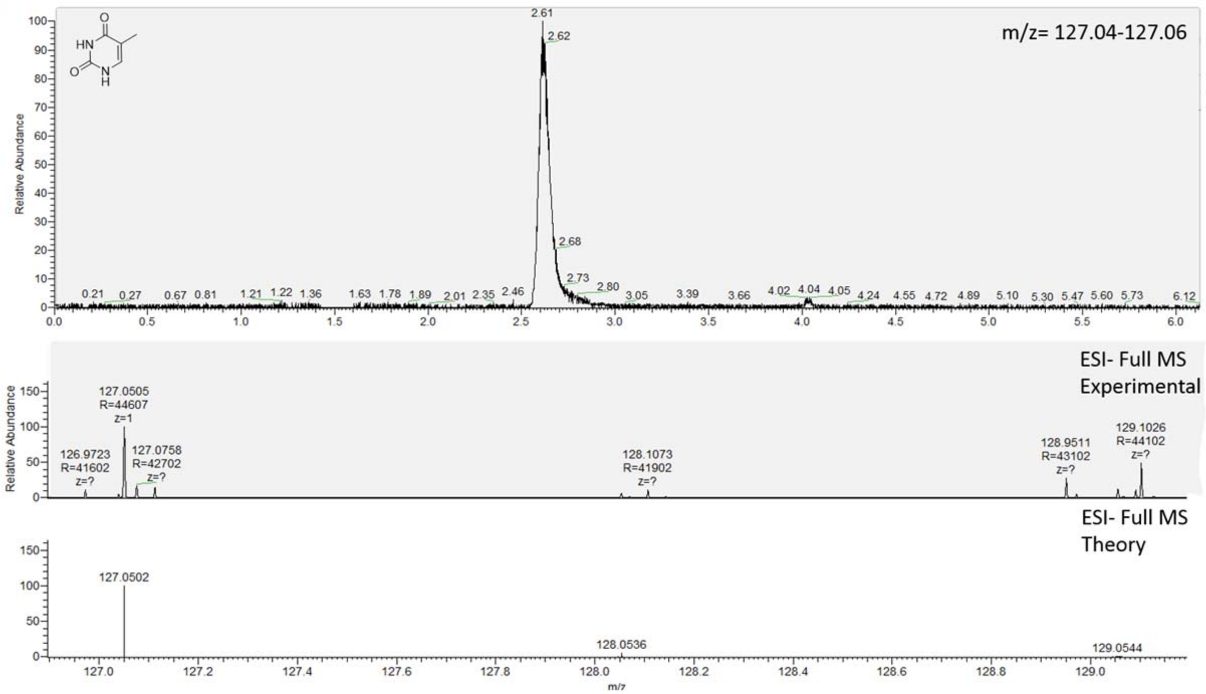

Supplementary Figure 32: Thy UHPLC-EIS/MS elution time (2.61 min), as well as the experimental and theoretical ESI-Full MS spectra. The parent ion peak here is found at 127.0505 m/z and is in good agreement with the theoretical ESI-Full MS peak at 127.0502 m/z. Here, a 250 nM sample was used along with 20 µL injection volumes.

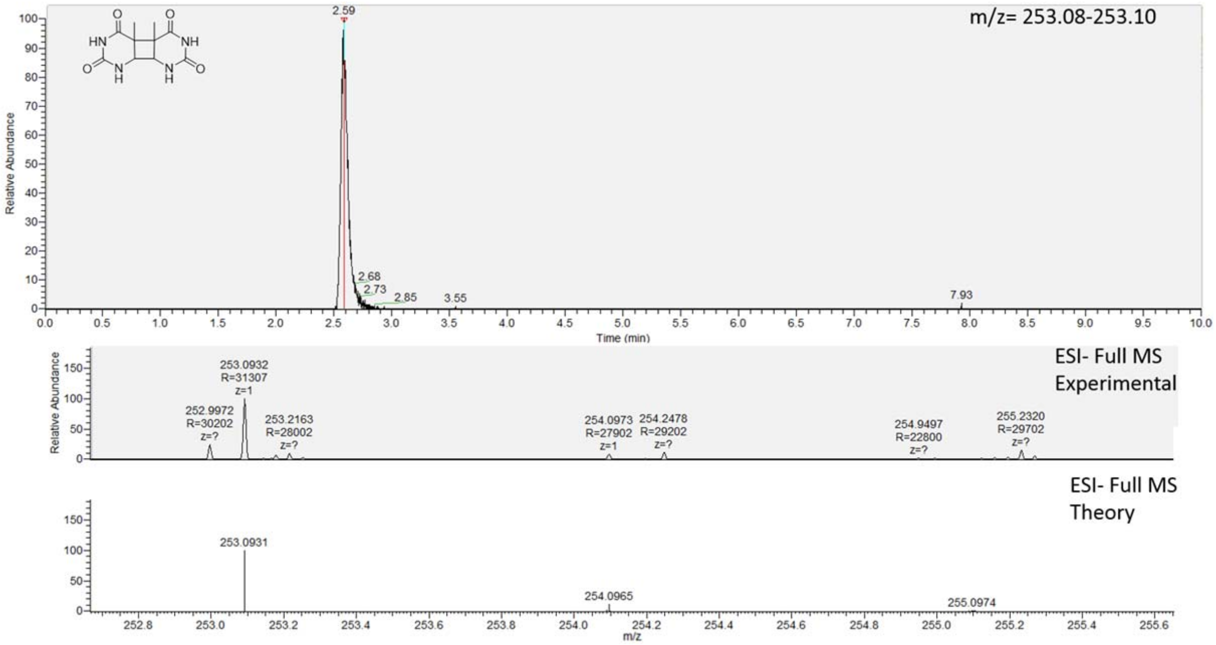

Supplementary Figure 33: Thy<>Thy UHPLC-EIS/MS elution time (2.59 min), as well as the experimental and theoretical ESI-Full MS spectra. The parent ion peak here is found at 253.0932 m/z and is in good agreement with the theoretical ESI-Full MS peak at 253.0931 m/z. Here, a 250 nM sample was used along with 30 µL injection volumes.

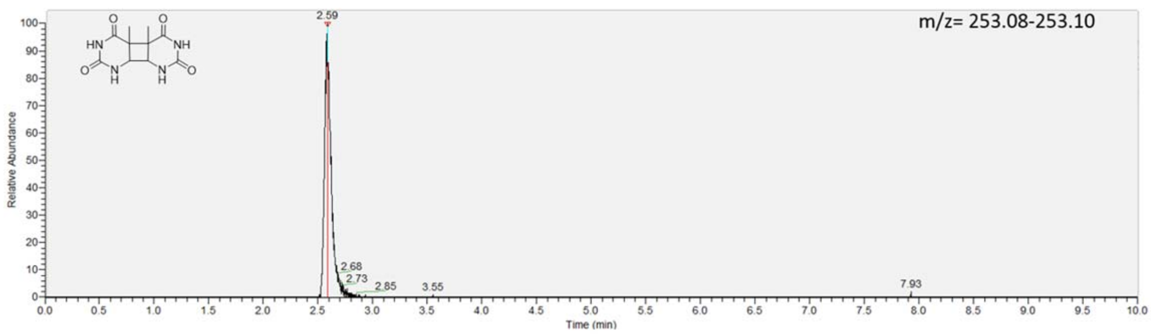

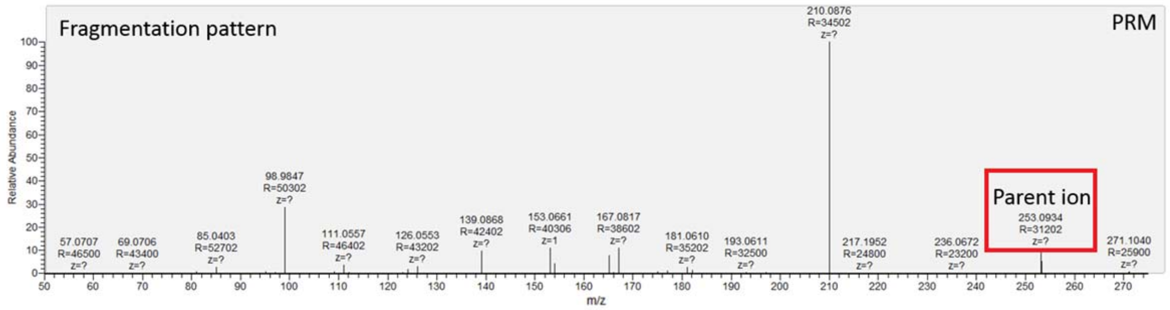

Supplementary Figure 34: Thy<>Thy UHPLC-EIS/MS elution time (2.59 min), and parallel reaction monitoring (PRM) to determine the fragmentation pattern. Two major fragments at 210.0676 m/z and 98.9847 m/z can be seen along with the parent ion (253.0934 m/z). These three peaks, along with the proper elution time, were later used to determine the presence of Thy<>Thy in each sample. Here, a 250nM sample was used along with 30 µL injection volumes.

Initial UHPLC-EIS/MS runs were performed on standards of both free Thy and Thy<>Thy to determine their elution times, parent ion peaks, and PRM fragmentation pattern which were later compared to samples of **SION-19@UV-Thy (20-80%),** which were exposed to UV (254 nm), and subsequently destroyed.

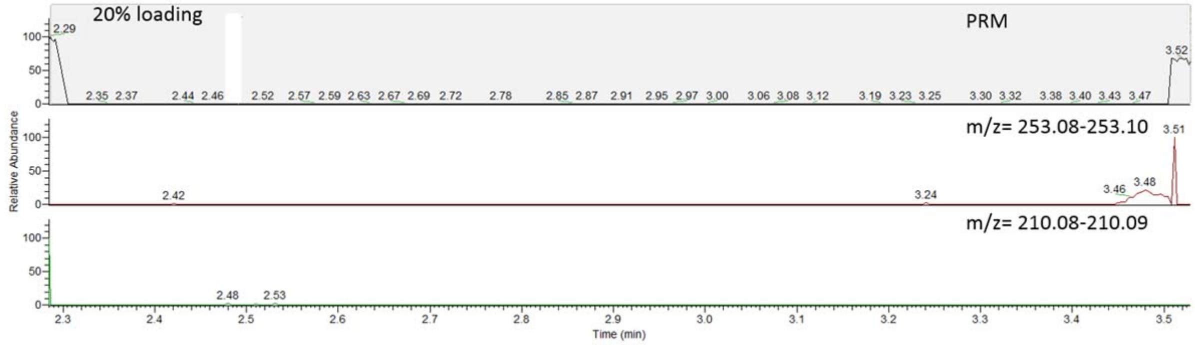
20% loading
PRM
m/z= 253.08-253.10
m/z= 210.08-210.09
Relative Abundance
Time (min)

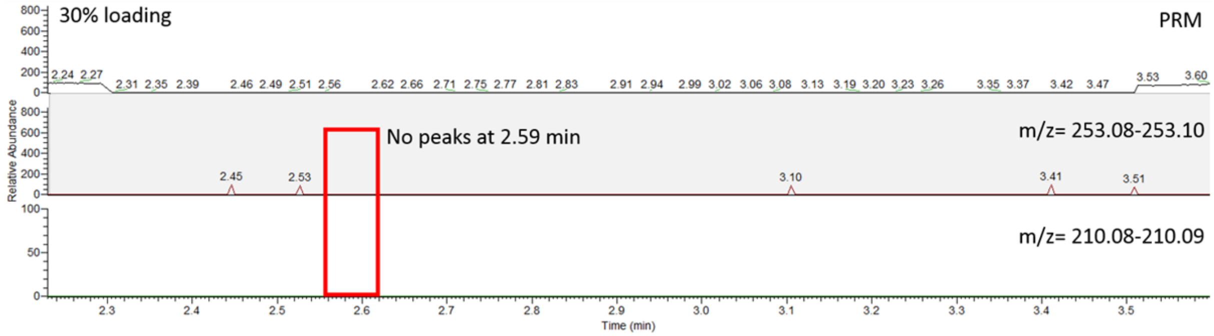
30% loading
PRM
No peaks at 2.59 min
m/z= 253.08-253.10
m/z= 210.08-210.09
Relative Abundance
Time (min)

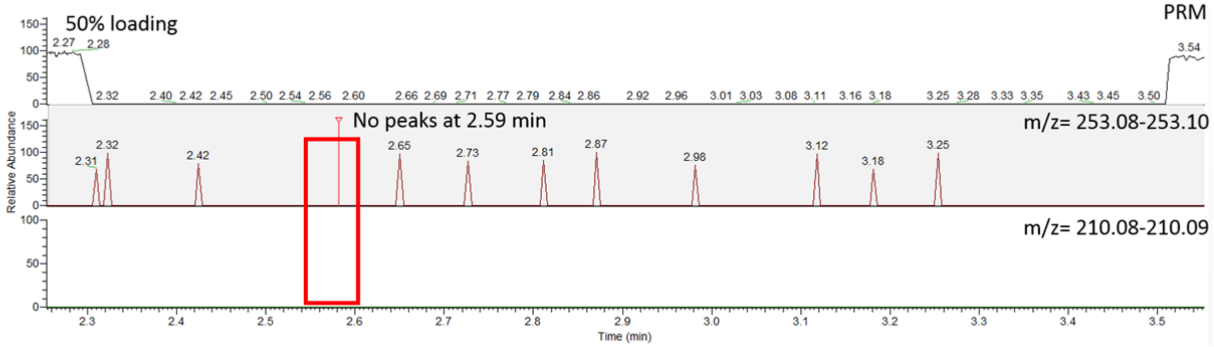
50% loading
PRM
No peaks at 2.59 min
m/z= 253.08-253.10
m/z= 210.08-210.09
Relative Abundance
Time (min)

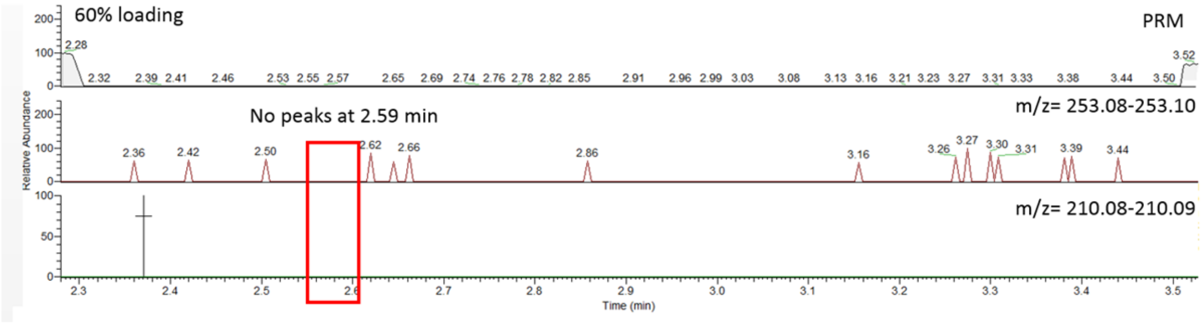
60% loading
PRM
No peaks at 2.59 min
m/z= 253.08-253.10
m/z= 210.08-210.09
Relative Abundance
Time (min)

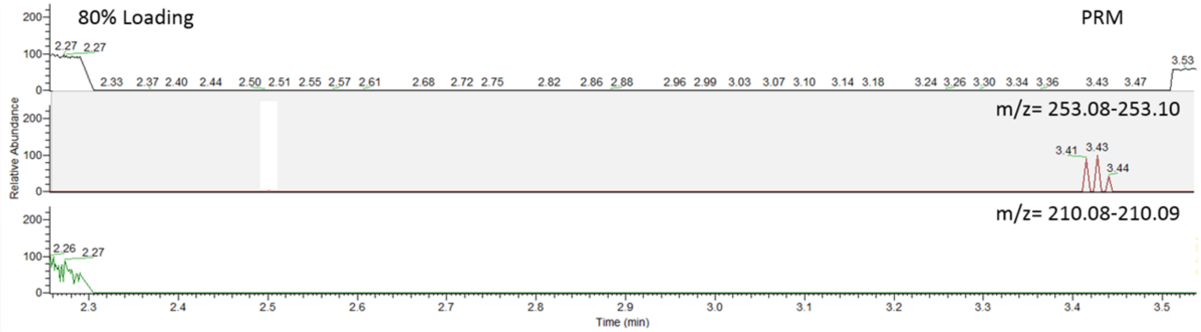

Supplementary Figure 35: SION-19@UV-Thy (20-30%, 50-80%) UHPLC-EIS/MS parallel reaction monitoring (PRM) between 2.3-3.5 min. No parent ion peaks were found.

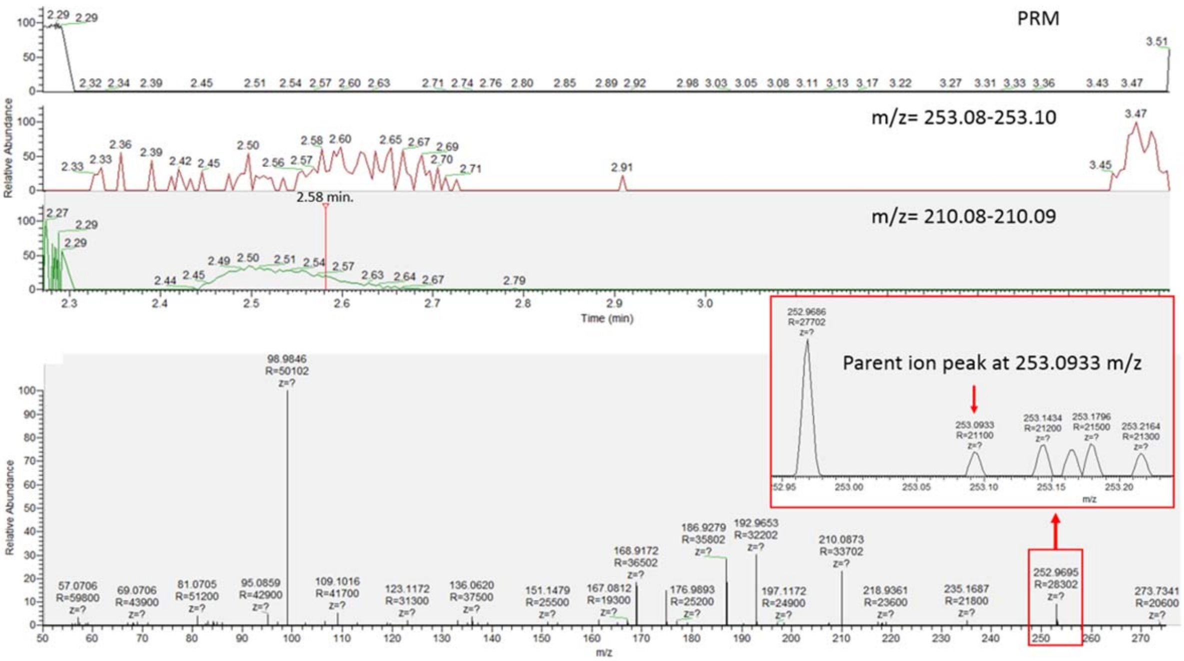

Supplementary Figure 36: SION-19@UV-Thy(40%), Trial 1: UHPLC-EIS/MS parallel reaction monitoring (PRM) between 2.3-3.5 min. searching for a mass of 253.08-253.10 afforded multiple peaks at different elution times. At an elution time of 2.58 min. a broad peak for 210.08-210.09 m/z can be seen, which corresponds to a fragmentation pattern of 253.0933 (parent ion), 210.0873 and 98.9846 m/z. Which are all in good agreement with the reference Thy<>Thy pattern with 253.0934 (parent ion), 210.0876 and 98.9847 m/z.

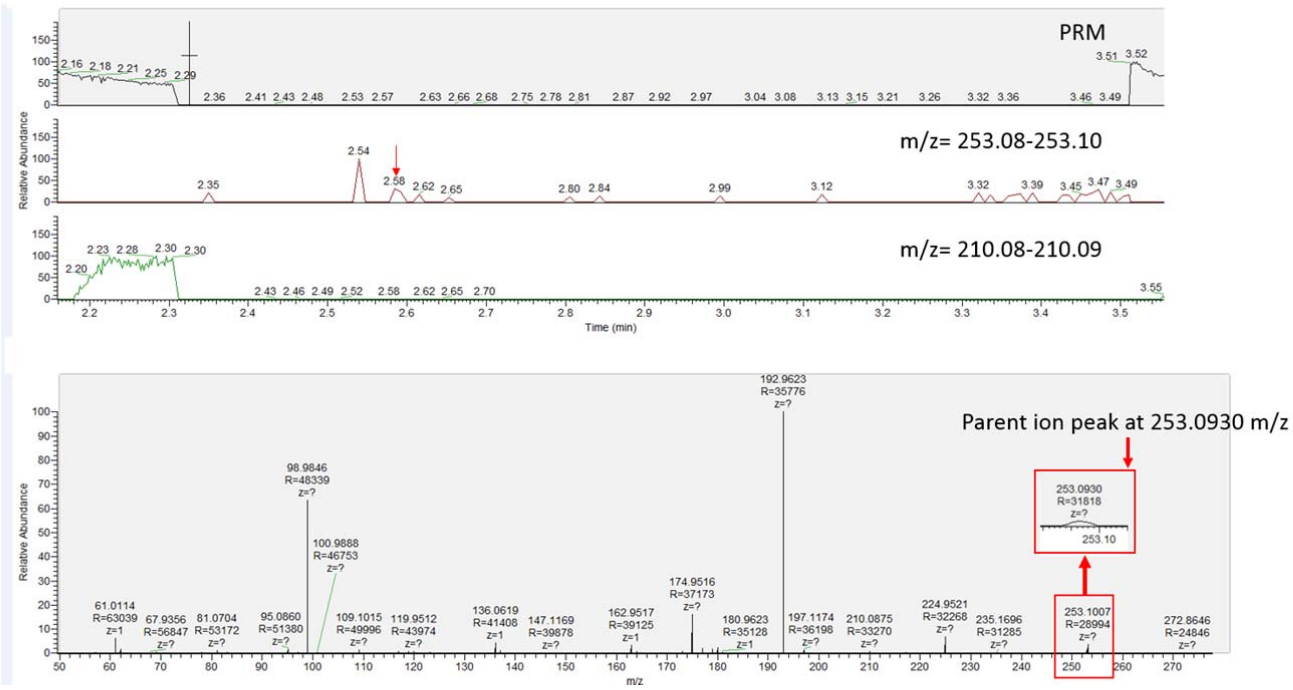

Supplementary Figure 37: SION-19@UV-Thy(40%), Trial 2: UHPLC-EIS/MS parallel reaction monitoring (PRM) between 2.3-3.5 min. searching for a mass of 253.08-253.10 afforded multiple peaks at different elution times. At an elution time of 2.58 min. a low broad peak for 210.08-210.09 m/z can be seen, which corresponds to a fragmentation pattern of 253.0930 (parent ion), 210.0875 and 98.9846 m/z. Which are all in good agreement with the reference Thy<>Thy pattern with 253.0934 (parent ion), 210.0876 and 98.9847 m/z.

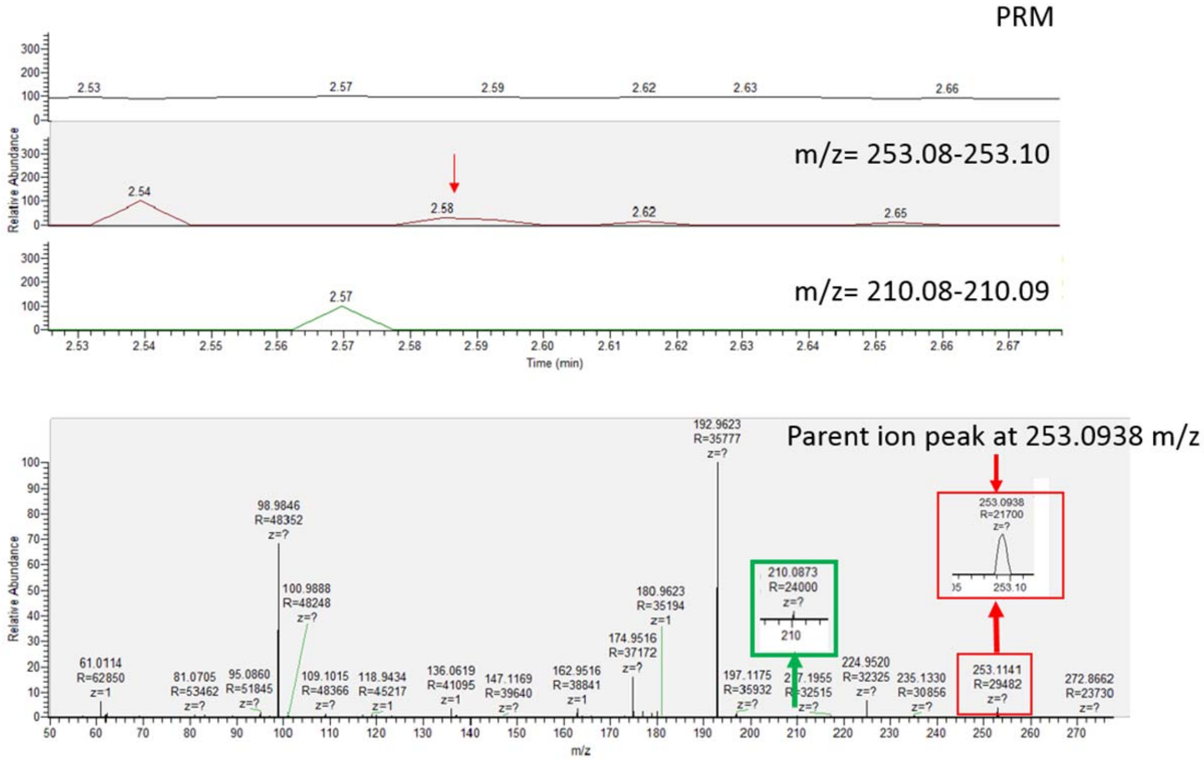

Supplementary Figure 38: SION-19@UV-Thy(40%), Trial 3: UHPLC-EIS/MS parallel reaction monitoring (PRM) between 2.3-3.5 min. searching for a mass of 253.08-253.10 afforded multiple peaks at different elution times. At an elution time of 2.58/2.57 min. a low broad peak for 210.08-210.09 m/z can be seen, which corresponds to a fragmentation pattern of 253.0938 (parent ion), 210.0873 and 98.9846 m/z. Which are all in good agreement with the reference Thy<>Thy pattern with 253.0934 (parent ion), 210.0876 and 98.9847 m/z.

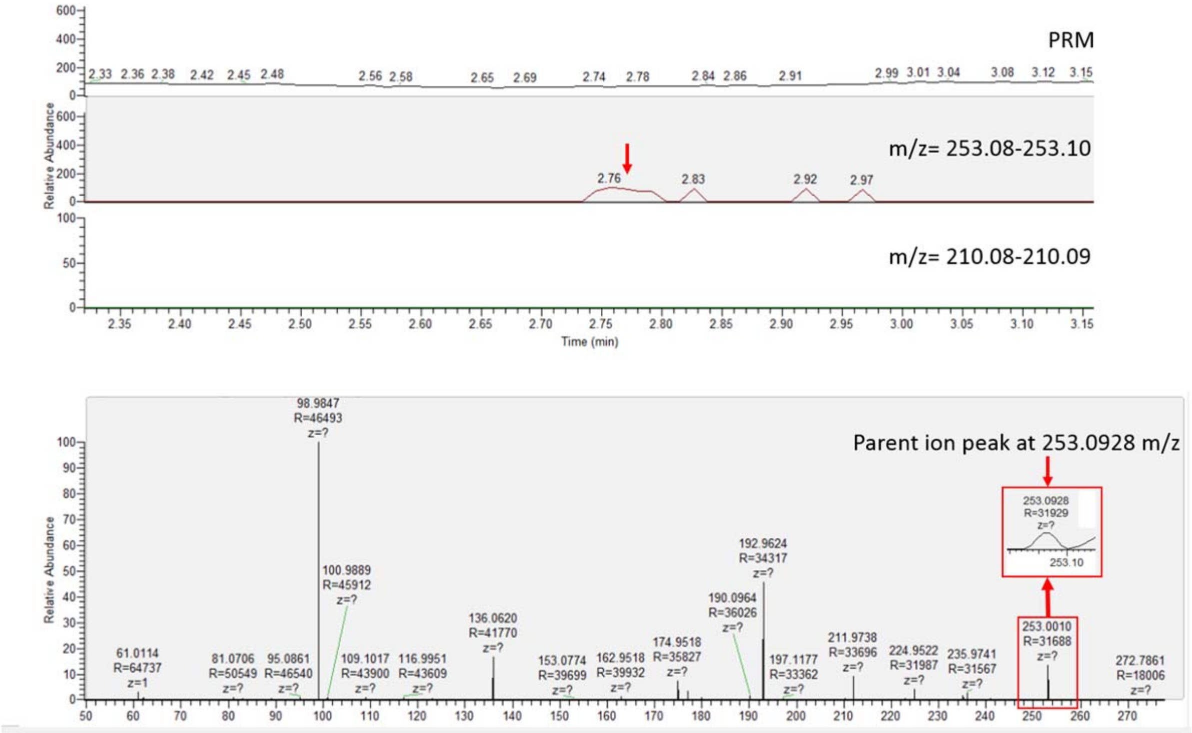

Supplementary Figure 39: SION-19@UV-Thy(45%), trial 1. UHPLC-EIS/MS parallel reaction monitoring (PRM) between 2.3-3.5 min. In the fragmentation pattern 253.0928 (parent ion), and 98.9847 m/z are in good agreement with the reference Thy<>Thy pattern with 253.0934 (parent ion), and 98.9847 m/z. Due to the low concentration of Thy<>Thy formation, a peak at 210.08 was not visible. It should be noted that the elution time of the Thy<>Thy molecules is shifted due to increased sample loading (40 µL of a sample containing 350 µL DMSO/Thy<>Thy in 1 mL of H_2O).

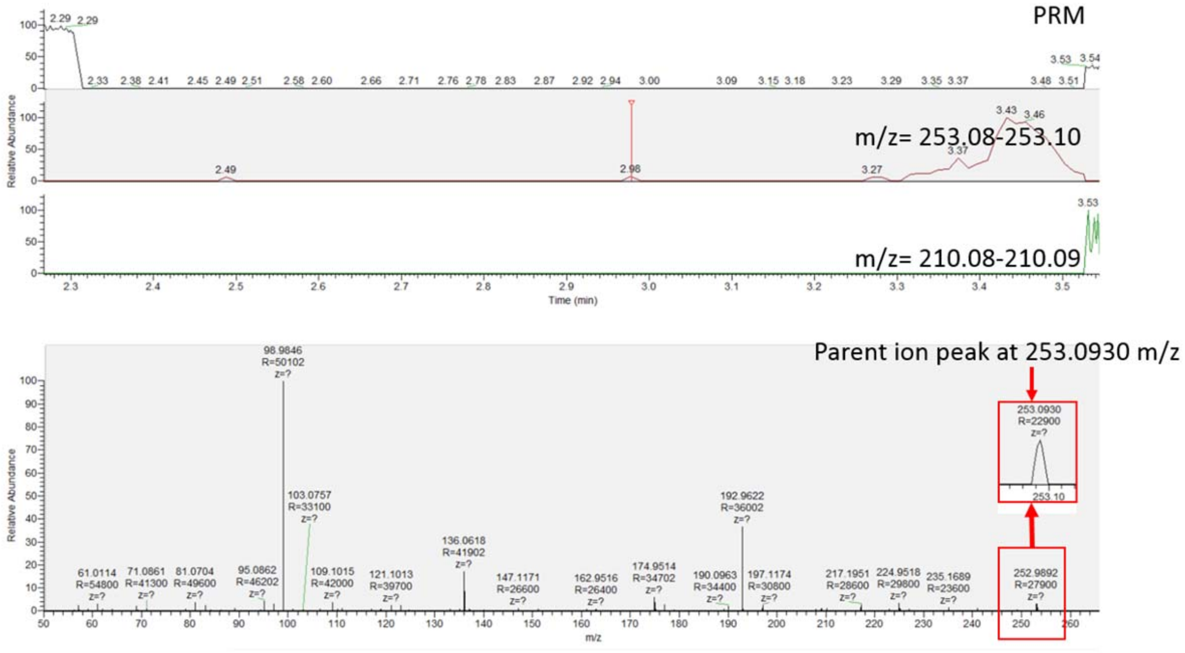

Supplementary Figure 40: SION-19@UV-Thy(45%), trial 2. UHPLC-EIS/MS parallel reaction monitoring (PRM) between 2.3-3.5 min. In the fragmentation pattern 253.0930 (parent ion), and 98.9846 m/z are in good agreement with the reference Thy<>Thy pattern with 253.0934 (parent ion), and 98.9847 m/z. It should be noted that the elution time of the Thy<>Thy molecules is shifted due to increased sample loading (50 µL of a sample containing 350 µL DMSO/Thy<>Thy in 1 mL of H_2O).

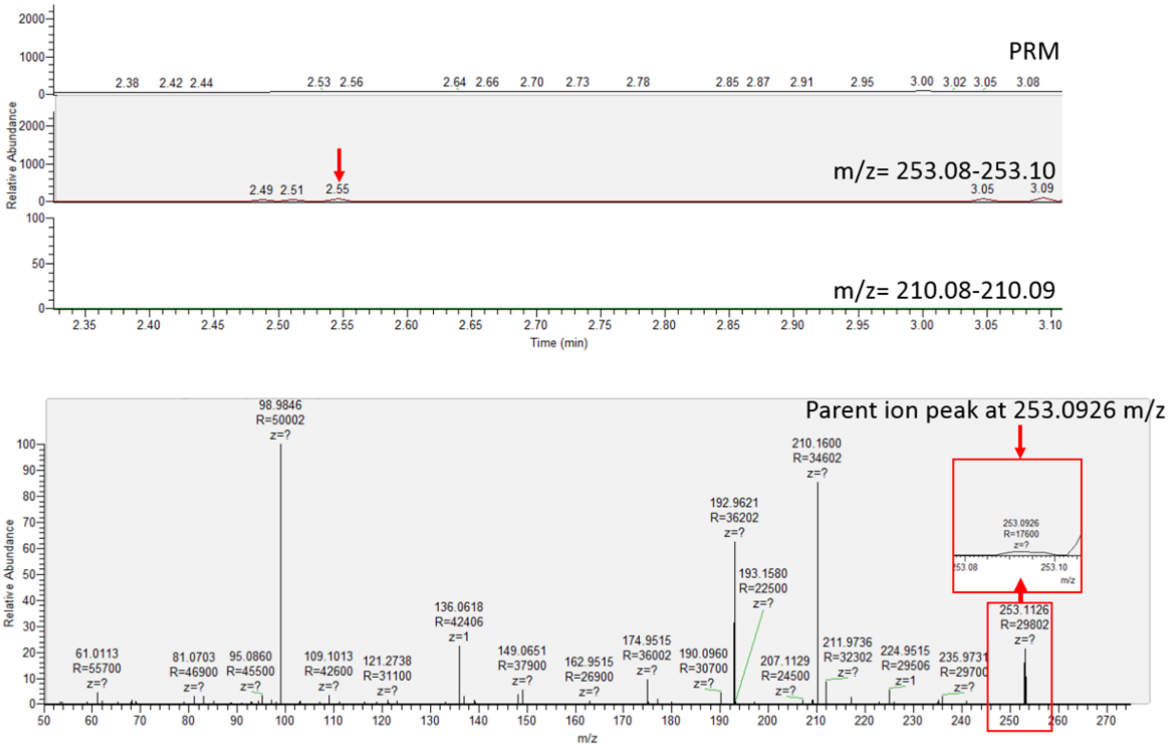

Supplementary Figure 41: SION-19@UV-Thy(45%), trial 3. UHPLC-EIS/MS parallel reaction monitoring (PRM) between 2.3-3.5 min. In the fragmentation pattern 253.0926 (parent ion), and 98.9846 m/z are all in good agreement with the reference Thy<>Thy pattern with 253.0934 (parent ion), and 98.9847 m/z.

Supplementary Discussion 8.4.1. Analysis for Unreacted Thy and Fragmentation Pattern for SION-19@UV-Thy(40%).

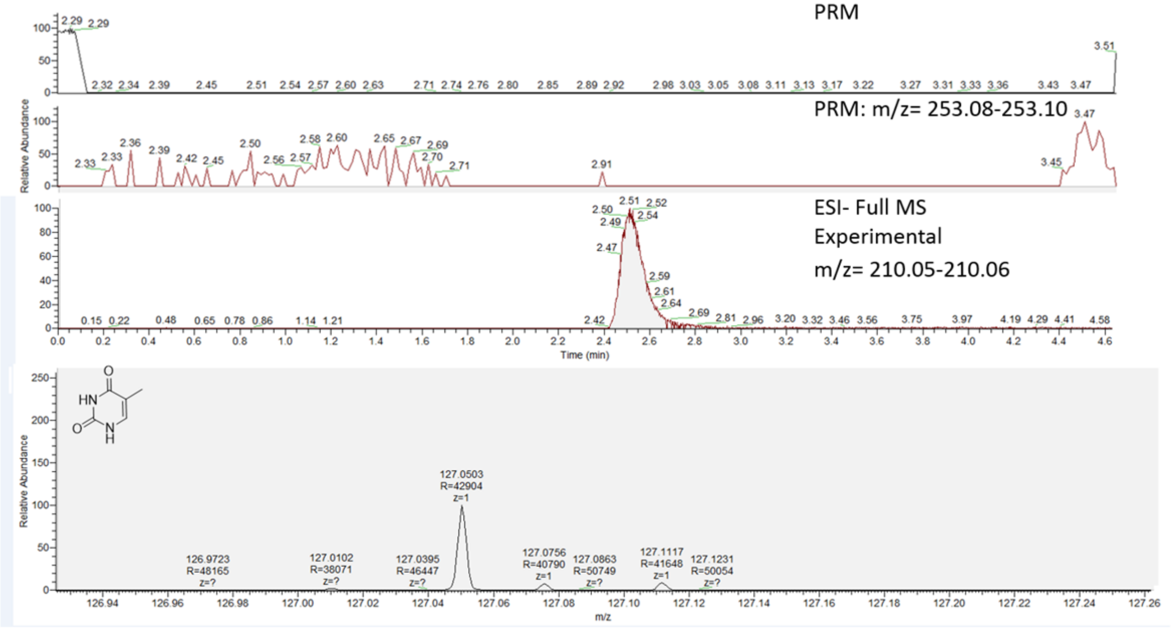

Supplementary Figure 42: SION-19@UV-Thy(40%) UHPLC-EIS/MS: Full MS and PRM between 2.3-3.5 min. Further analysis of **SION-19@UV-Thy(40%)** for unreacted Thy yields a parent ion peak of 127.0503 m/z which is in agreement with the observed Thy parent ion peak of 127.0505 m/z and theoretical parent ion peak of 127.0502 m/z.

Supplementary Table 9: Summary of UHPLC-EI/MS for **SION-19@UV-Thy(20-80%)**, Thy, and Thy<>Thy. It should be noted for Trials 1 and 2 of **SION-19@UV-Thy(45%)** higher concentrations of sample were required to see the parent ion.

	**Elution time (min)**	**Theoretical parent ion (m/z)**	**Experimental parent ion (m/z)**	**Common fragments (i.e. base peak, etc.) (m/z)**
**Thy**	2.61	127.0502	127.0505	-----
**Thy<>Thy**	2.59	253.0931	253.0932	210.0876,<br>98.9847
**SION-19@UV-Thy (20%)**	-----	-----	-----	-----
**SION-19@UV-Thy (30%)**	-----	-----	-----	-----
**SION-19@UV-Thy (40%)**	2.58	-----	Trial 1: 253.0933<br>Trial 2: 253.0930<br>Trial 3: 253.0938	Trial 1: 210.0873<br>98.9846<br>Trial 2: 210.0875<br>98.9846<br>Trial 3: 210.0873<br>98.9846<br><br>Unreacted Thy (127.0503 m/z)
**SION-19@UV-Thy (45%)**	Trial 1: 2.76<br>Trial 2: 2.98<br>Trial 3: 2.55		Trial 1: 253.0928<br>Trial 2: 253.0930<br>Trial 3: 253.0926	Trial 1: 98.9847<br>Trial 2: 98.9846<br>Trial 3: 98.9846<br><br>Unreacted Thy (127.0503 m/z)
**SION-19@UV-Thy (50%)**	-----	-----	**-----**	-----
**SION-19@UV-Thy (60%)**	-----	-----	**-----**	-----
**SION-19@UV-Thy (80%)**	-----	-----	-----	-----

Supplementary Discussion 8.4.2. Isolation of Thy<>Thy from SION-19@UV-Thy(40%).

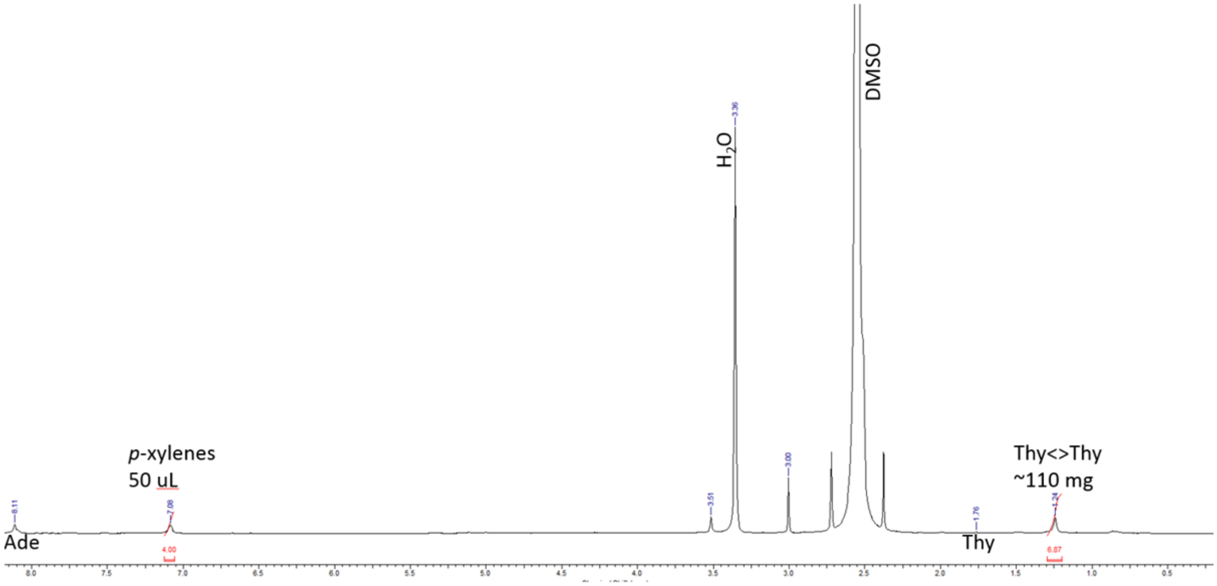

Supplementary Figure 43: The **SION-19@UV-Thy(40%)** was used for the isolation of Thy<>Thy for the conversion calculations.

Using the same method previously described, 500 mg of **SION-19'** was loaded with Thy (40%) and irradiated with UV light (254 nm). The MOF was subsequently dissolved with 5 mL of a 0.5 M K_2CO_3 solution. The solution was then concentrated using a rotary evaporator, and loaded onto a column using H_2O. Here, H_2O removes the majority of the TBAPy ligand which is a salt. The column is flushed with water, and rinsed with $CHCl_3$ to remove any trace of H_2O. The residual left in the beaker is loaded onto the column with DMSO, and flushed through the column with approximately 150 mL of DMSO in total. The DMSO eluent is concentrated using a rotary evaporator, and loaded into an NMR tube with $_d$-DMSO. Using *p*-xylenes as an internal standard (50 µL) the approximate amount of Thy<>Thy is observed to be 110 mg (Figure S43).

Since one formula unit of **SION-19** is $[Zn_{1.5}O_{0.25}(Ade)(TBAPy)_{0.5}]\cdot(NH_2Me_2)_{0.5}$, and a 40 % loading of Thy was used, a 100% yield of Thy<>Thy would be 0.00079292 moles Thy<>Thy. From the NMR internal standard, 110 mg of Thy<>Thy yields 0.00046432 mol, and a 58.5 % conversion rate.

Supplementary Discussion 9. Bio-MOF-1, HKUST-1 and Zn-MOF-74: Thy to Thy<>Thy Dimerization within the Pores.

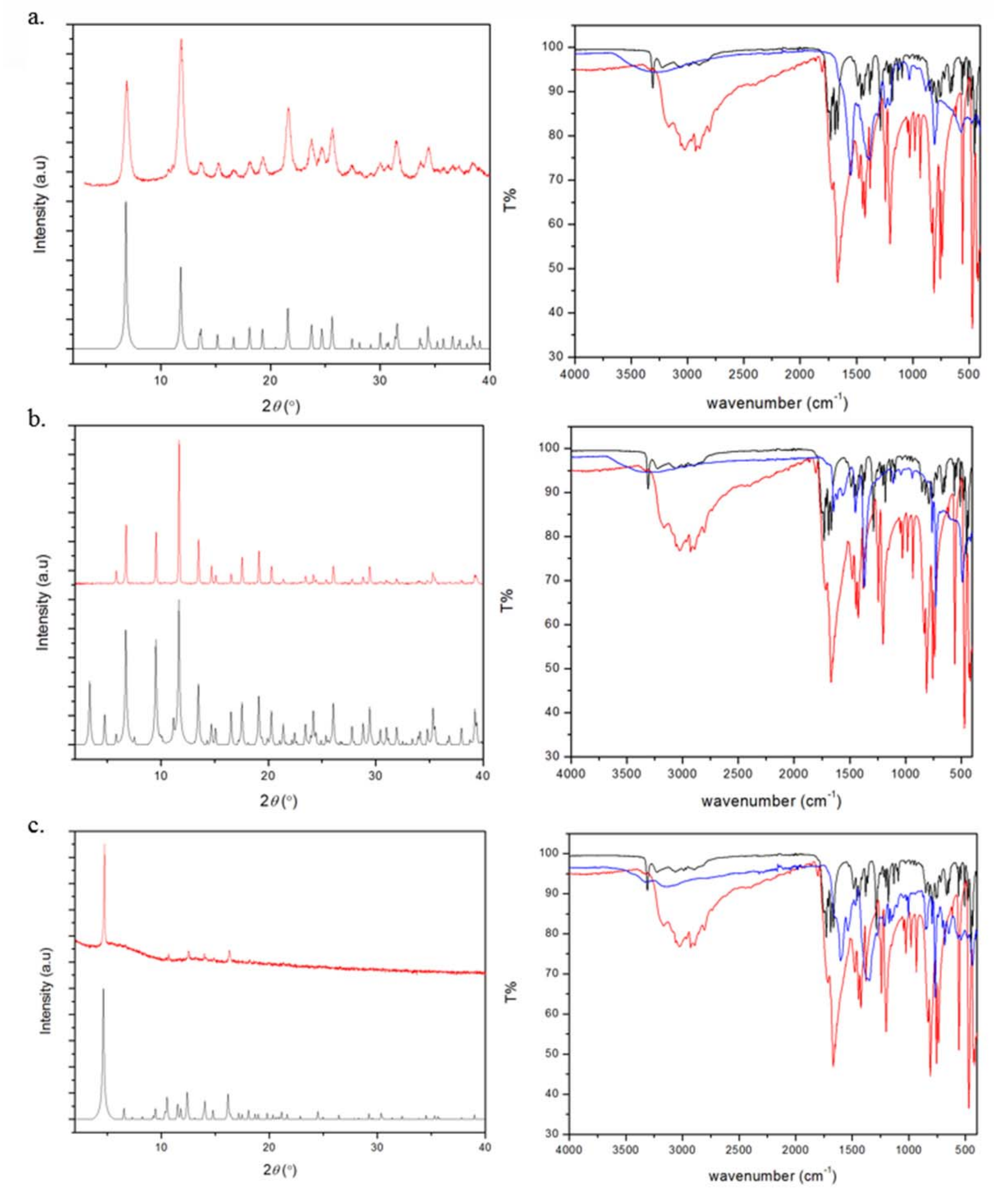

Supplementary Figure 44: a) left: **Zn-MOF-74** PXRD. Color scheme: black, theory; red, **Zn-MOF-74**. Right: IR of Zn-MOF-74. Color scheme: red, Thy; black, Thy<>Thy; blue, **Zn-MOF-74**. b) left: **HKUST-1** PXRD. Color scheme: black, theory; red, **HKUST-1**. Right: IR of Zn-MOF-74. Color scheme: red, Thy; black, Thy<>Thy; blue, **HKUST-1**. c) left: **Bio-MOF-1** PXRD. Color scheme: black, theory; red, **Bio-MOF-1**. Right: IR of Zn-MOF-74. Color scheme: red, Thy; black, Thy<>Thy; blue, **Bio-MOF-1**.

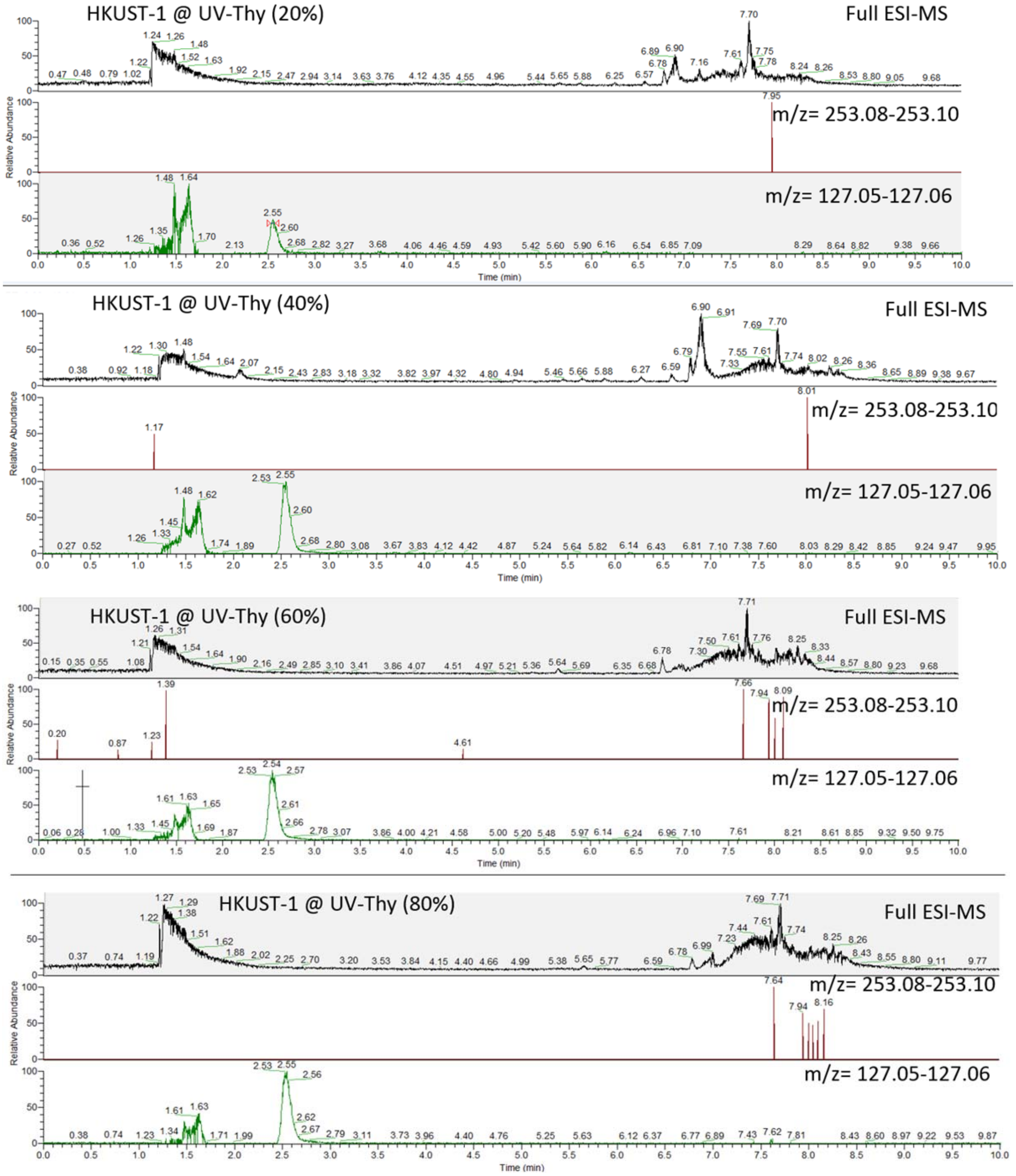

Supplementary Figure 45: **HKUST-1@UV-Thy (20-80%)** HPLC-EIS/MS. In all experiments, no peak at 2.59 min is found for a mass between 253.08-253.10 corresponding to Thy<>Thy. Instead, only a peak at 2.54-2.55 min is found with m/z = 127.05-127.06 is found, which corresponds to Thy. From this analysis, it can be concluded that Thy does not dimerize within the pores of HKUST-1.

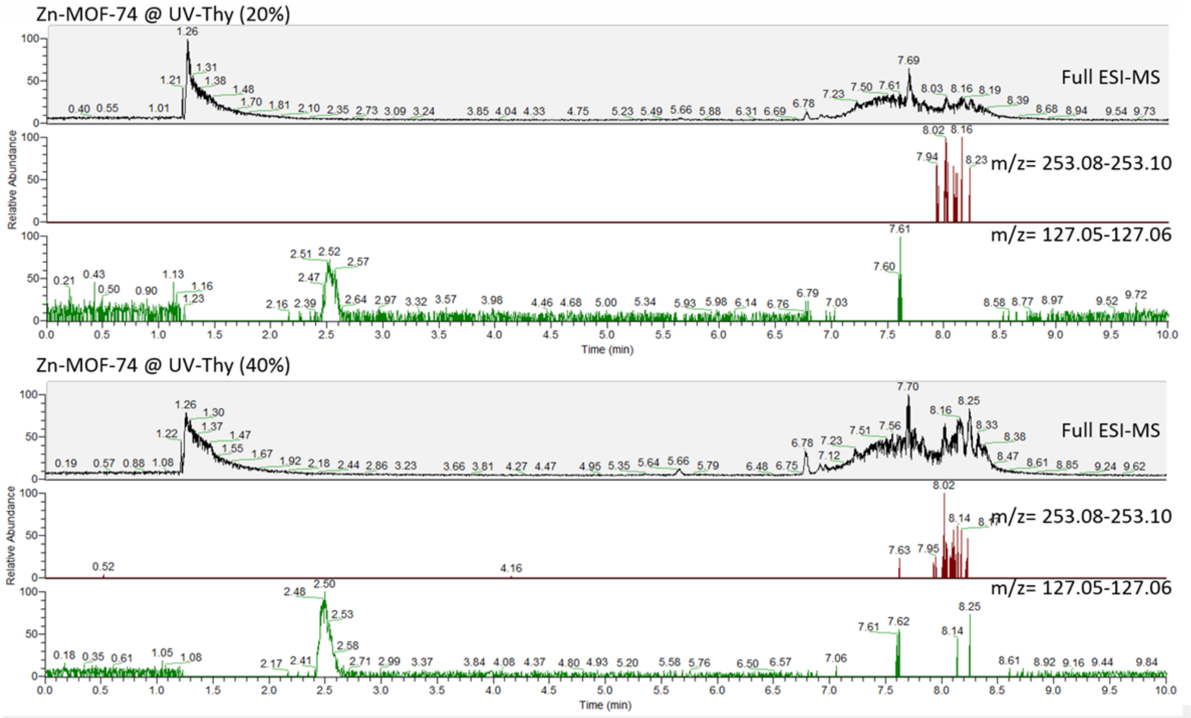

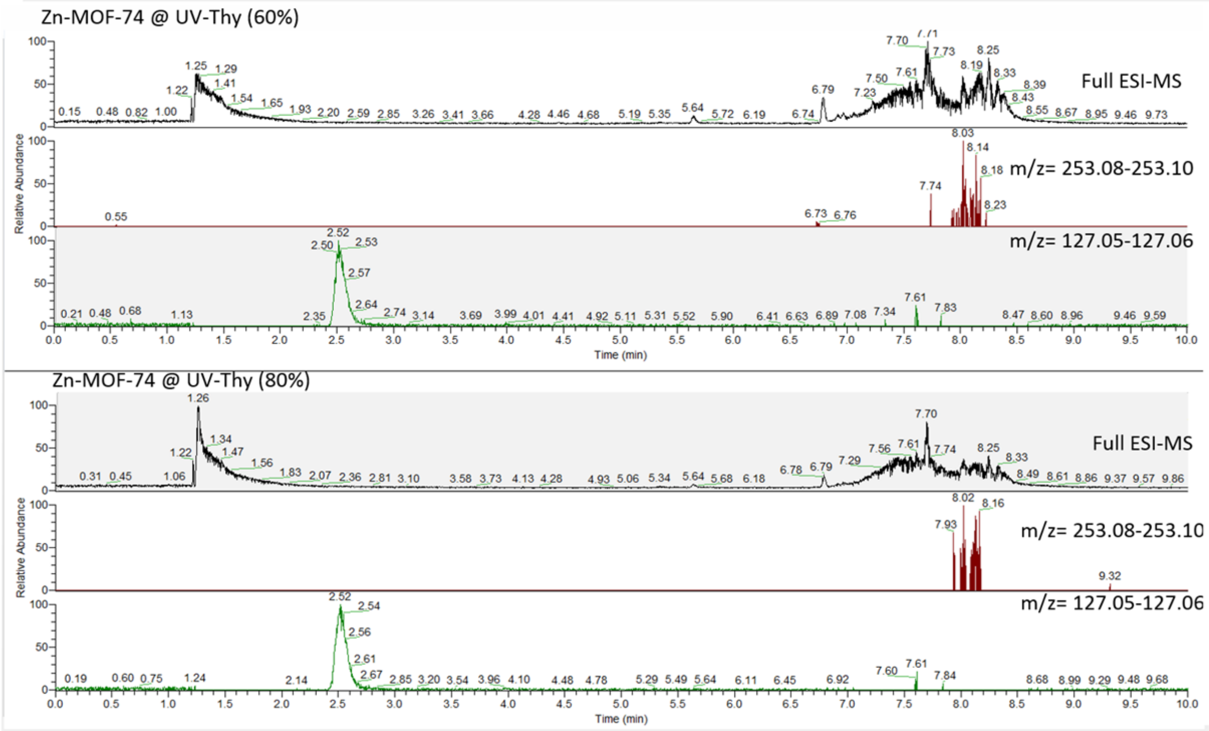

Supplementary Figure 46: **Zn-MOF-74@UV-Thy(20-80%)** HPLC-EIS/MS. In all experiments, no peak at 2.59 min is found for a mass between 253.08-253.10 corresponding to Thy<>Thy. Instead, only a peak at 2.54-2.55 min is found with m/z = 127.05-127.06 is found, which corresponds to Thy. From this analysis, it can be concluded that Thy does not dimerize within the pores of Zn-MOF-74.

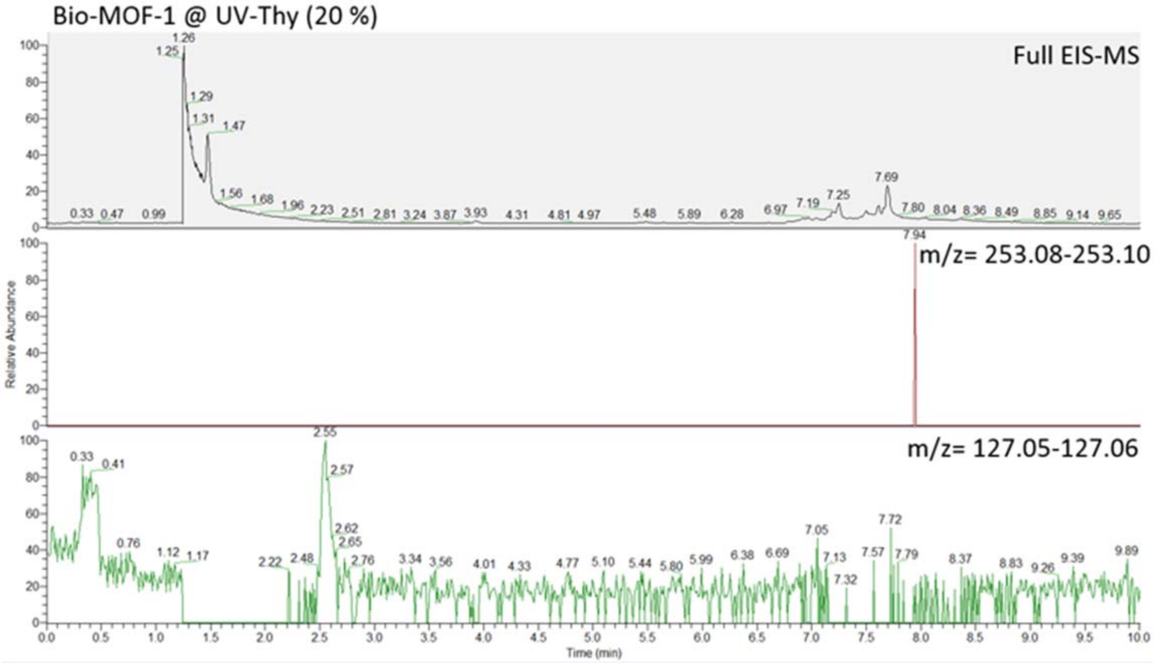

Bio-MOF-1 @ UV-Thy (20 %)
Full EIS-MS
m/z= 253.08-253.10
m/z= 127.05-127.06
Relative Abundance
Time (min)

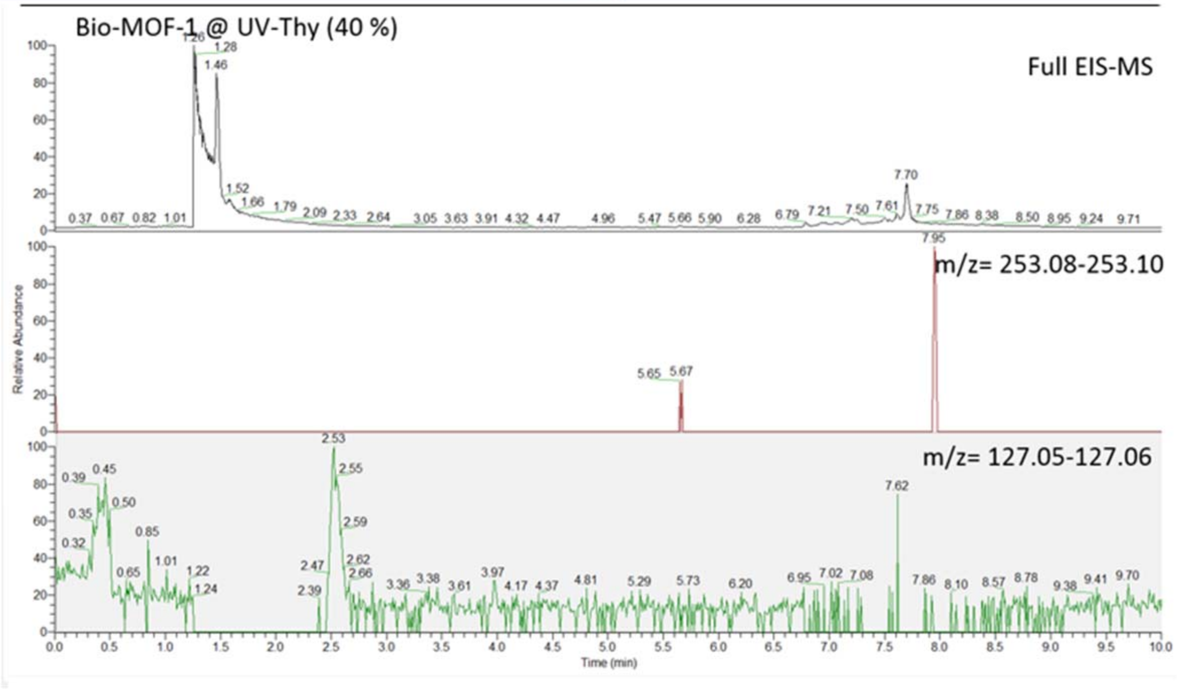

Bio-MOF-1 @ UV-Thy (40 %)
Full EIS-MS
m/z= 253.08-253.10
m/z= 127.05-127.06
Relative Abundance
Time (min)

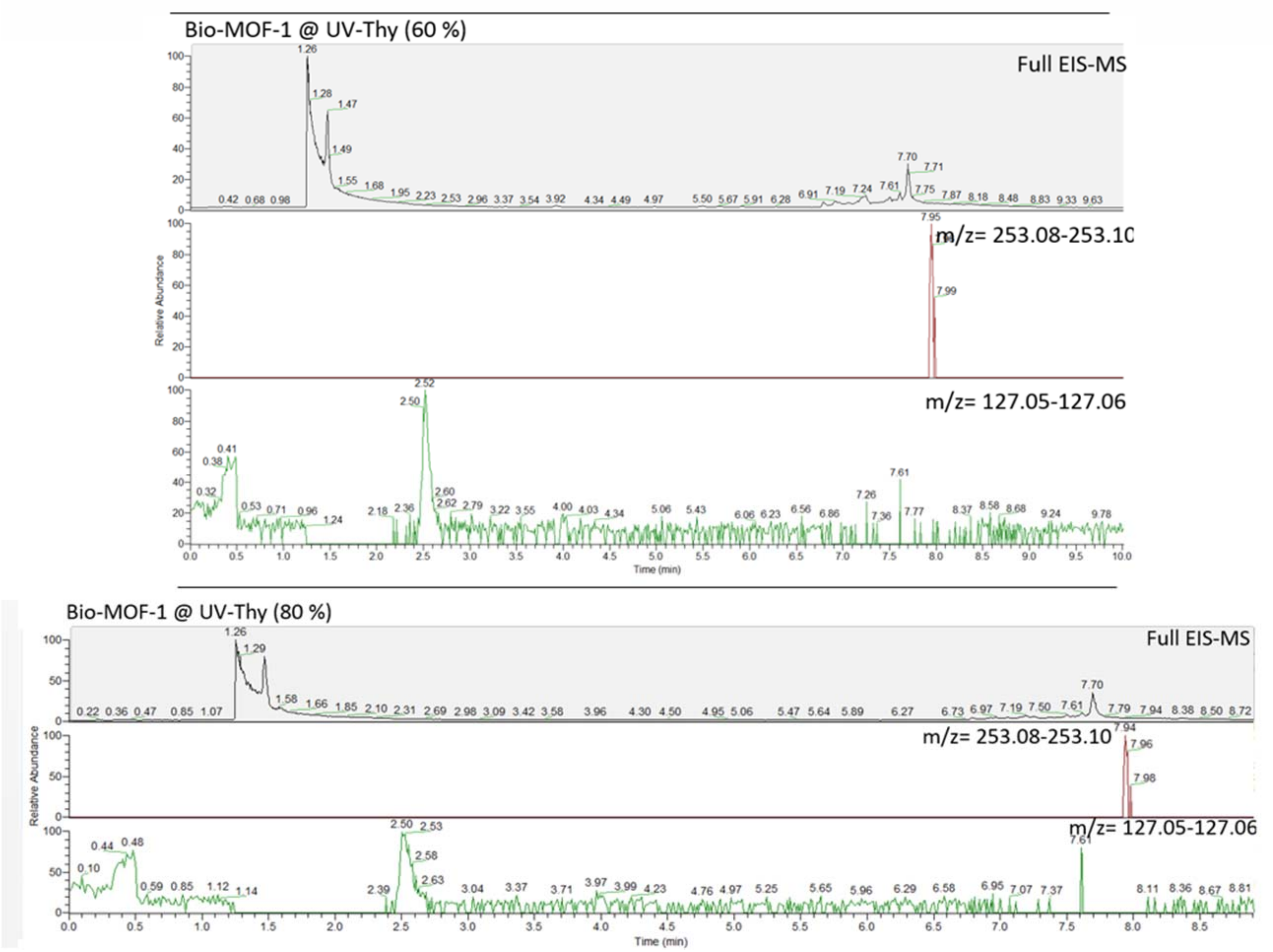

Supplementary Figure 47: **Bio-MOF-1@UV-Thy(20-80%)** HPLC-EIS/MS. In all experiments, no peak at 2.59 min is found for a mass between 253.08-253.10 corresponding to Thy<>Thy. Instead, only a peak at 2.54-2.55 min is found with m/z = 127.05-127.06 is found, which corresponds to Thy. From this analysis, it can be concluded that Thy does not dimerize within the pores of **Bio-MOF-1**.

10. Supplementary References.

1. Rigaku, O. (Oxford Diffraction Ltd, Yarnton, 2015).
2. Sheldrick, G. Crystal structure refinement with SHELXL. *Acta Crystallographica Section C* **71**, 3-8 (2015).
3. Palatinus, L. & Chapuis, G. SUPERFLIP–a computer program for the solution of crystal structures by charge flipping in arbitrary dimensions. *J. Appl. Crystallogr.* **40**, 786-790 (2007).
4. Palatinus, L. & van der Lee, A. Symmetry determination following structure solution in P1. *J. Appl. Crystallogr.* **41**, 975-984 (2008).
5. Palatinus, L., Prathapa, S.J. & Smaalen, S.v. EDMA: a computer program for topological analysis of discrete electron densities. *J. Appl. Crystallogr.* **45**, 575-580 (2012).
6. Dolomanov, O.V., Bourhis, L.J., Gildea, R.J., Howard, J.A.K. & Puschmann, H. OLEX2: a complete structure solution, refinement and analysis program. *J. Appl. Crystallogr.* **42**, 339-341 (2009).
7. Van der Sluis, P. & Spek, A. BYPASS: an effective method for the refinement of crystal structures containing disordered solvent regions. *Acta. Cryst.* **46**, 194-201 (1990).
8. Blatov, V.A., Shevchenko, A.P. & Proserpio, D.M. Applied Topological Analysis of Crystal Structures with the Program Package ToposPro. *Cryst. Growth Des.* **14**, 3576-3586 (2014).
9. Feyer, V. et al. Bonding at the organic/metal interface: Adenine to Cu(110). *Phys. Rev. B.* **79**, 155432 (2009).
10. Kresse, G. & Furthmüller, J. Efficient iterative schemes for ab initio total-energy calculations using a plane-wave basis set. *Phys. Rev. B.* **54**, 11169-11186 (1996).
11. Kresse, G. & Furthmüller, J. Efficiency of ab-initio total energy calculations for metals and semiconductors using a plane-wave basis set. *Comput. Mater. Sci.* **6**, 15-50 (1996).
12. Kresse, G. & Hafner, J. Ab initio. *Phys. Rev. B.* **49**, 14251-14269 (1994).
13. Kresse, G. & Hafner, J. Ab initio. *Phys. Rev. B.* **47**, 558-561 (1993).
14. Perdew, J.P., Burke, K. & Ernzerhof, M. Generalized Gradient Approximation Made Simple [Phys. Rev. Lett. 77, 3865 (1996)]. *Phys. Rev. Lett.* **78**, 1396-1396 (1997).
15. Perdew, J.P., Burke, K. & Ernzerhof, M. Generalized Gradient Approximation Made Simple. *Phys. Rev. Lett.* **77**, 3865-3868 (1996).
16. Blöchl, P.E. Projector augmented-wave method. *Phys. Rev. B.* **50**, 17953-17979 (1994).
17. Ongari, D. et al. Accurate Characterization of the Pore Volume in Microporous Crystalline Materials. *Langmuir* **33**, 14529-14538 (2017).
18. Campañá, C., Mussard, B. & Woo, T.K. Electrostatic Potential Derived Atomic Charges for Periodic Systems Using a Modified Error Functional. *J. Chem. Theory Comput.* **5**, 2866-2878 (2009).
19. Plimpton, S. Fast Parallel Algorithms for Short-Range Molecular Dynamics. *J. Comput. Phys.* **117**, 1-19 (1995).

20. Mayo, S.L., Olafson, B.D. & Goddard, W.A. DREIDING: a generic force field for molecular simulations. *J. Phys. Chem.* **94**, 8897-8909 (1990).
21. Liu, Y., Bryantsev, V.S., Diallo, M.S. & Goddard I, W.A. PAMAM Dendrimers Undergo pH Responsive Conformational Changes without Swelling. *J. Am. Chem. Soc.* **131**, 2798-2799 (2009).
22. Willems, T.F., Rycroft, C.H., Kazi, M., Meza, J.C. & Haranczyk, M. Algorithms and tools for high-throughput geometry-based analysis of crystalline porous materials. *Micropor. Mesopor. Mat.* **149**, 134-141 (2012).
23. Pinheiro, M. et al. Characterization and comparison of pore landscapes in crystalline porous materials. *J. Mol. Graph. Model.* **44**, 208-219 (2013).
24. Pinheiro, M., Martin, R.L., Rycroft, C.H. & Haranczyk, M. High accuracy geometric analysis of crystalline porous materials. *CrystEngComm* **15**, 7531-7538 (2013).
25. Michaud-Agrawal, N., Denning, E.J., Woolf, T.B. & Beckstein, O. MDAnalysis: A toolkit for the analysis of molecular dynamics simulations. *J. Comput. Chem.* **32**, 2319-2327 (2011).
26. J Gowers, R. et al. MDAnalysis: a Python package for the rapid analysis of molecular dynamics simulations. (2016).
27. Füchtbauer, W. & Mazur, P. Kinetics of the ultraviolet-induced dimerization of thymine in frozen solutions*. *J. Photochem. Photobiol.* **5**, 323-335 (1966).
28. Beukers, R. & Berends, W. The effects of u.v.-irradiation on nucleic acids and their components. *Biochim. Biophys. Acta* **49**, 181-189 (1961).
29. Cadet, J. et al. Characterization of thymidine ultraviolet photoproducts. Cyclobutane dimers and 5,6-dihydrothymidines. *Can. J. Chem.* **63**, 2861-2868 (1985).
30. Burdi, D., Hoyt, S. & Begley, T.P. Design of a cleavable linker for the synthesis of a cis-syn pyrimidine photodimer. *Tetrahedron Lett.* **33**, 2133-2136 (1992).
